# Extracellular Vesicles: An Emerging Mechanism Governing the Secretion and Biological Roles of Tenascin-C

**DOI:** 10.3389/fimmu.2021.671485

**Published:** 2021-04-26

**Authors:** Lucas Albacete-Albacete, Miguel Sánchez-Álvarez, Miguel Angel del Pozo

**Affiliations:** Mechanoadaptation and Caveolae Biology Lab, Area of Cell and Developmental Biology, Centro Nacional de Investigaciones Cardiovasculares (CNIC), Madrid, Spain

**Keywords:** tenascin C, extracellular matrix (ECM), exosomes, fibronectin (FN), tumor progression, cardiovascular disease, inflammation

## Abstract

ECM composition and architecture are tightly regulated for tissue homeostasis. Different disorders have been associated to alterations in the levels of proteins such as collagens, fibronectin (FN) or tenascin-C (TnC). TnC emerges as a key regulator of multiple inflammatory processes, both during physiological tissue repair as well as pathological conditions ranging from tumor progression to cardiovascular disease. Importantly, our current understanding as to how TnC and other non-collagen ECM components are secreted has remained elusive. Extracellular vesicles (EVs) are small membrane-bound particles released to the extracellular space by most cell types, playing a key role in cell-cell communication. A broad range of cellular components can be transported by EVs (e.g. nucleic acids, lipids, signalling molecules and proteins). These cargoes can be transferred to target cells, potentially modulating their function. Recently, several extracellular matrix (ECM) proteins have been characterized as *bona fide* EV cargoes, exosomal secretion being particularly critical for TnC. EV-dependent ECM secretion might underpin diseases where ECM integrity is altered, establishing novel concepts in the field such as ECM nucleation over long distances, and highlighting novel opportunities for diagnostics and therapeutic intervention. Here, we review recent findings and standing questions on the molecular mechanisms governing EV–dependent ECM secretion and its potential relevance for disease, with a focus on TnC.

## Introduction

Multicellularity drove the emergence of cell differentiation and functional specialization, changing the continuous communication cells establish with their surrounding environment. A connective substance among tissues ensuring nurturing and functional coordination between cells evolved, giving rise to the extracellular matrix (ECM) (1, 2). In addition to providing a physical scaffold, the ECM actively participates of several biochemical and biomechanical processes related to morphogenesis, differentiation and homeostasis. A meshwork generally composed of water, proteins, glycoproteins and proteoglycans, the ECM exhibits tissue-specific matrix composition and architecture, which provide unique physicochemical properties (3, 4). Importantly, the ECM is constantly remodelled by cells to maintain tissue and organismal homeostasis across conditions (5–7). Apart from the regulated secretion of specific structural components, ECM architectural remodeling is orchestrated by secreted modifying enzymes (metalloproteinases (MMPs) (8) and their inhibitors (TIMPs) (9), and other enzymes controlling ECM modification and crosslinking—such as lysyl oxidases (LOX) (10) or transglutaminases—, and a reciprocal biomechanical crosstalk with resident cells (11). Several growth factors and cytokines are bound to the ECM and modulate cell adhesion, differentiation, growth and migration (12) and its architecture and physical properties can modulate cell function (13). Conversely, cell proliferation, spatial arrangement and contractility drives ECM remodeling (14–16).

The broad functional relevance of the ECM is reflected by the numerous pathological conditions associated with ECM alteration or dysfunction. Some of these diseases are related to genetic abnormalities that imply a decrease in the expression, or post-translational modification, of certain ECM proteins (17–20). On the other hand, desmoplasia—an increase in bulk ECM deposition and/or dysregulated expression of certain ECM components—(21), causes architectural and biomechanical alterations driving different pathologies, including cardiovascular diseases, chronic inflammation or cancer.

Tenascins are a family of extracellular matrix (ECM) glycoproteins composed of five members (Tenascin-C (TnC), R, W, X and Y), TnC being the best characterized among them (22). TnC is a hexameric protein which contributes to regulate cell substrate adhesion through the modulation of focal adhesion (FA) binding to other ECM components such as fibronectin (FN) (23), and downstream events such as cell activation, apoptotic cascades, and migration. TnC is expressed abundantly during development, especially in the neural system. However, expression levels of TnC in adults are substantially reduced and its presence is virtually limited to stem cell niches and tendons. Increased TnC expression in adult, differentiated tissues is commonly associated with tissue damage and repair, as well as with pathological conditions such as dysregulated inflammation (as occurs, for example, in atherosclerotic lesions) or tumorigenesis (24–31).

Despite their physiopathological relevance, our understanding of the intracellular mechanisms regulating the trafficking and secretion of TnC and many other ECM components is limited (32). Notably, recent studies support that extracellular vesicles (EVs), including exosomes and microvesicles (MVs), can act as carriers of ECM components, including TnC and FN, a well-known, evolutionarily related partner of TnC (33, 34). Here, we review our current knowledge on the role of EVs on TnC secretion and ECM deposit, and their potential relevance for inflammation and disease.

## Physiopathological Roles of TnC and Their Molecular Basis

Certain features of tumor progression and metastasis are currently considered subverted, aberrant wound repair programs (35), where ECM deposit and remodeling by resident fibroblast is dysregulated. This altered stromal ECM can in turn promote several cancer hallmarks (36). For example, sustained proliferation requires cell adhesion to ECM and growth factor-dependent activation of Erk and PI3K, to promote G1/S transition. The ECM can also promote the induction of hypoxia-triggered angiogenesis acting as a reservoir of angiogenesis regulators, activate cell invasion through the regulation of cell adhesion and invadopodia formation, or modulate the immune response (13, 37). Several ECM components exhibiting differential expression and/or arrangement in tumors play relevant roles in the progression of the disease. Altered deposition of different collagen types can regulate cell growth, differentiation and cell migration. An excessive deposition of collagen I in many solid tumor types confers rigidity to the tumor stroma, and its altered assembly and crosslinking, mechanical properties and architectural features such as anisotropy, affect tumor cell biology (3, 5). Other key ECM components also exhibit altered expression in cancer. FN is considered a major building block in ECM fibre assembly and remodeling, and can bind to other molecules including heparin, collagens, tenascins or fibrin to modulate their assembly and their interaction with cells (38, 39).

During development, TnC is expressed robustly and contributes to physiological epithelial-to-mesenchymal transitions (EMT) and morphogenesis (25). In contrast, in normal adult tissues TnC expression is usually low, except for stem cell niches and tendons. Upon tissue damage, TnC can be rapidly upregulated and contributes to physiological inflammation and repair. Owing to its capacity to promote proinflammatory and activated states in different cell types, increased TnC deposition is associated with several pathological conditions. Persistent high levels of TnC can promote chronic inflammation and desmoplasia, driving pathological events such as fibrosis or oncogenesis.

TnC was initially characterized as a modulator of cell adhesion, either through its interaction with other ECM components (23) or through direct binding to specific cell receptors. Its binding to integrins such as α9β1, αVβ3, α8β1 and αVβ6 (27, 28) can induce EMT in several cancer models (40–42), modulate the dynamics of focal adhesions (43, 44) or reduce apoptosis. These characteristics support its potential as a marker of poor prognosis, underpinned by its impact on cell motility and invasion, aberrant angiogenesis (45) and immunomodulation (31, 46, 47). Importantly, TnC modulates the activation state of immune cells such as macrophages and lymphocytes; this appears to be an important aspect of its contribution to both physiological tissue repair, as well as pathological conditions involving tissue remodeling (48–50).

Several mouse models reveal the importance of TnC in tumor progression and its implication in tumor cell survival, proliferation, invasion and metastasis (51, 52). TnC can influence fibroblasts and differentiation of epithelial cells onto myofibloblasts through the tumor growth factor-β (TGF-β) signalling pathway (53), regulate inflammatory signalling by an activation of Toll-like receptor 4 (TLR4) (54) or modulate epidermal growth factor (EGF)-receptor driven cell proliferation cell proliferation (55). As part of the AngioMatrix (56), TnC can participate in the angiogenic switch, and generate an aberrant vasculature within tumours. Both as a result of this promotion of aberrant angiogenesis, as well as through direct modulation of immune cell populations, TnC is likely an important contributor to the emerging role of stromal ECM composition and architecture as central regulators of antitumor immunity (57–60). An intriguing feature that may be particularly relevant for the rationalization of TnC as a biomarker, or even therapeutic target, in the context of antitumor immunotherapy is its potential to selectively determine macrophage polarity towards M1-like, cytokine-releasing phenotypes (mainly through its interaction with α9β1, αVβ3 and TLR4 receptors); and promote an anergic state in T-cells (presumably by interfering with integrin signalling) (61, 62). Recent studies have shown the beneficial effects of targeting TnC in antitumor immunotherapy in breast (63) and oral squamous cell carcinoma (64) mouse models. Combinational therapy with monoclonal antibodies that inhibited TnC-mediated TLR4 activation and anti-PD-L1 treatment significantly reduced tumor growth and lung metastasis *in vivo* (63). In line with these results, ablation of TnC or its effector CCR7 implied inhibition of the lymphoid immune-suppressive stromal properties, reducing tumor progression and metastasis in oral squamous cell carcinoma (64), indicating a relevant approach in the therapy of head and neck tumors.

TnC has a prominent role in cardiovascular tissue remodeling. Almost invariably, TnC re-expression is associated with cardiovascular pathological processes coursing with inflammation, such as myocardial infarction, hypertensive cardiac fibrosis, myocarditis or dilated cardiomyopathy (65–67). Upregulation of TnC is also a hallmark of the proatherogenic vessel remodeling, driving the progression of atherosclerotic disease (AS) (68–70); however, TnC deficiency in mouse models of genetic hypercholesterolemia exacerbate atherosclerosis and promote lesions prone to rupture, reflecting the delicate balance between the physiological roles of TnC in tissue homeostasis (71).

TnC can play a role in several diseases derived from a fibrotic state generated upon tissue damage (50, 52, 72, 73). For example, in neuroinflammation (29), brain injury (74) or glioma (59), where ECM deposition is enhanced, TnC accumulation is found associated with blood-brain barrier disruption, neuronal apoptosis and activation of inflammatory pathways (mitogen-activated protein kinases and NF-kB). Finally, TnC is implicated in other fibrotic diseases such as kidney and liver damage through orchestration of the fibrotic niche and is considered as a biomarker of poor prognosis (75, 76).

## The Standing Question of TnC Secretion

**Figure 1:**
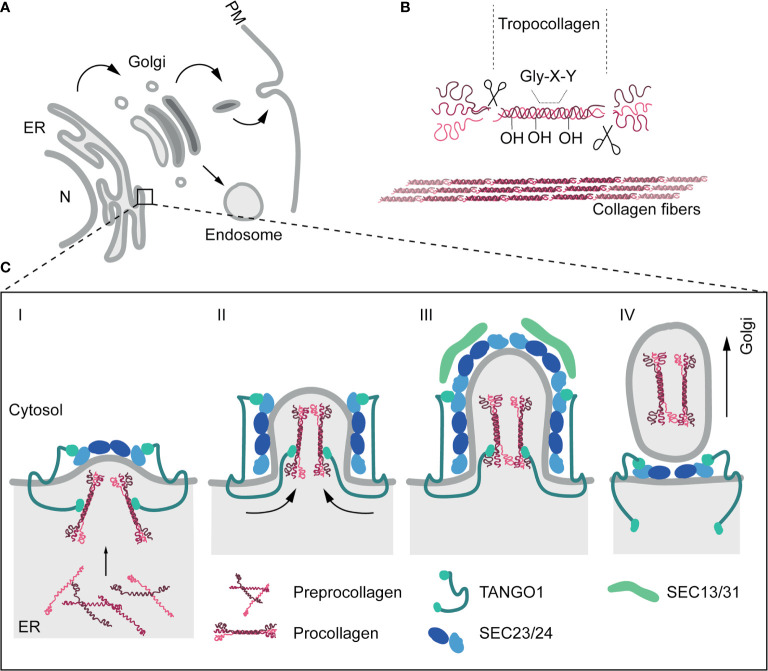
The secretory pathway and collagen secretion. **(A)** Schematic representation of cell secretion routes. The ER constitutes the main protein factory in the cell. ER-associated ribosomes translate proteins that can be subsequently inserted onto the membrane, or released into the ER lumen. After translation, several modifications can be added to proteins bydifferent enzymatic activities. Proteins are then transported to the Golgi apparatus, mainly through the COPII-dependent pathway. Within the Golgi, further modifications are carried out. Finally, proteins will be sorted into vesicles and transported to their final destination, including the plasma membrane (PM) (receptors, adhesion proteins and extracellular proteins) or endosomes. **(B)** Collagens are initially secreted as procollagens. Once in the extracellular space, terminal peptides are cleaved by the procollagen peptidase to form tropocollagen. Finally, collagen fibrils are assembled *via* covalent cross-linking by lysyl oxidases, which link hydroxylysine and lysine residues. Multiple collagen fibrils assemble into collagen fibers. **(C)** In addition to COPII machinery, other regulators are necessary for the proper export of procollagen from the ER. TANGO1 participates in the sorting of procollagen in vesicles through a direct binding through Hsp47 in the lumen of the ER. Preprocollagen peptides are synthesised and assembled in the lumen of the ER. Once procollagens are formed, TANGO1, previously recruited trough the interaction to Sec23 (Sec23/24 complex), position the collagen fibrils in the budding vesicle (Stage I). Later, as the vesicle grow, TANGO1 pushes procollagen molecules towards the lumenal face of the ER (Stage II). In stage III, ER vesicles are big enough to accommodate collagens. TANGO1 separates its SH3-like domain from Hsp47/collagens. This is the followed by the release of TANGO1 from Sec23 and the recruitment of Sec13/31 to the ER membrane. Finally, in stage IV, fission of the collagen-containing vesicles is undertaken and TANGO1 return to interact with the Sec23/24 complex (84).

ECM component biogenesis, intracellular trafficking and export pathways are tightly controlled, but our current mechanistic understanding of these processes, particularly regarding non-collagen ECM components, is rather limited. Collagens, a family of large fibrillar ECM proteins, constitute over 30% of the total protein mass in multicellular organisms (77–79). These proteins are initially synthesized as an immature form, known as preprocollagen, in the endoplasmic reticulum (ER). These polypeptides undergo hydroxylation of proline and lysine residues and are assembled as triple helices, yielding procollagens (80). Procollagens must then be trafficked to the Golgi apparatus for further posttranslational modification. The coat complex type II (COPII) vesicle transport machinery facilitates the regulated transfer of proteins from the ER to the ER-Golgi intermediate compartment (ERGIC) and cis-Golgi (81, 82), and is strictly required for procollagen trafficking and secretion: mutation or genetic ablation of core COPII components such as SAR1B, SEC23A, SEC24A/C or SEC13 profoundly affect the secretion of collagens and lead to their accumulation in the ER (32). In contrast with smaller cargoes, procollagen units are too big (~300nm in length) to be incorporated into conventional ~80-nm COPII (83), and additional regulators (transport and Golgi organization protein 1 (TANGO1), cutaneous T-cell lymphoma-associated antigen 5 (cTAGE5), trafficking From ER To Golgi Regulator (TFG), or the KHLH12-cullin-3 ubiquitin E3 ligase complex) (84–87) have been identified as required for nascent COPII vesicles to accommodate and carry these rigid fibrillar molecules. Finally, procollagens are transported in tubular structures emanating from the Golgi to the plasma membrane (PM) and secreted to the extracellular space, where they will be cleaved to generate tropocollagens and assembled in crosslinked fibrils (**Figure 1**).

While this canonical route is relatively well characterized for collagens, several of its regulators, including core components of the COPII machinery, appear to be dispensable for the secretion of other ECM components. Indeed, the mechanisms governing the trafficking and secretion of a majority of non-collagen ECM proteins have remained puzzlingly elusive (32, 88). Soluble FN, which assembles in fibrillar structures upon secretion to the extracellular space and binding exposed integrins (38), is initially synthesized in the ER (32, 89). Current models describe its transport to the extracellular space through the secretory pathway (90–93), as it reaches the Golgi apparatus (94–98) to undergo further glycosylation (39). However, FN secretion seems to be unaffected by mutation or genetic ablation of core COPII components that severely impair collagen transport from the ER, such as SEC23A (99), SEC24D (100) or TANGO1 (32, 101), and its trafficking remains incompletely explored. Proteins such as periostin (89) and transmembrane P24 Trafficking Protein 2 (TMED2; the human homolog of *emp24*) (102) are proteins potentially associated with the export of FN from the ER.

TnC has a six arm-structure termed hexabrachion, consisting of six 320kDa monomers stabilized by amino-terminal disulphide bonds. In contrast to FN, oligomerization of TnC is a rapid process that takes place cotranslationally in the ER, and two models have been proposed. In one model, the six monomers are simultaneously assembled into a single hexabrachion, as suggested by pulse-chase approaches which found no apparent intermediate species (103). In the second model, oligomerization is a two-step process (104), whereby two intermediate trimers are first formed through the stabilization of alpha-helical coiled-coil interactions at their amino-terminal domains. Then, hexabrachion assembly is favoured by an increase in homophylic binding affinity between the two trimers. Similar to FN, evidence supporting TnC transit through (105), and glycosylation at (24), the Golgi, suggests that TnC is trafficked from the ER to the Golgi apparatus. This transfer appears to be a rate-limiting step for secretion output (103) and is affected in cells treated with brefeldin-A, an inhibitor of ER-Golgi vesicle transport (106). However, like for many other ECM components, the precise mechanisms regulating TnC trafficking and secretion remain incompletely characterized. An unexpected, emerging mechanism for the secretion of these and other non-collagen components, is extracellular vesicle (EV) secretion.

## EV Biogenesis and General Functions

Recent studies show that extracellular vesicles (EVs) can export ECM components to the extracellular environment (107), constituting alternative mechanisms for ECM secretion and deposition and implying specific regulatory principles for their trafficking (108, 109). EVs are a heterogeneous group of cell-derived membranous structures that include exosomes and MVs, which defer on their intracellular origin (110).

Exosome biogenesis takes place in the endosomal compartment through endosome membrane budding (111–113). Several mechanisms have been implicated in this process. One of the most studied mechanisms is dependent on the ESCRT (Endosomal Sorting Complexes Required for Transport) machinery, whose four conserved complexes (ESCRT-0, -I, -II and -III) (114–116) assemble sequentially on the cytosolic surface of the endosomal membrane. Ubiquitylation is an important event not only for ESCRT-dependent vesicle formation, but also for the specification of cargo to be sorted onto exosomes (113, 117). Additionally, evidence for an ESCRT-independent mechanism for exosome biogenesis has been described (118). Specific lipid species such as ceramides (derived from sphingomyelinases-mediated hydrolysis of sphingomyelin) (119), LBPA (lyso-bis-phosphatidic acid) (120) or cholesterol, as well as proteins that modulate membrane organization, including tetraspanins (121) and caveolin-1 (108, 122, 123), have been recently identified as important regulators of ESCRT-independent endosome dynamics and exosome biogenesis.

**Figure 2:**
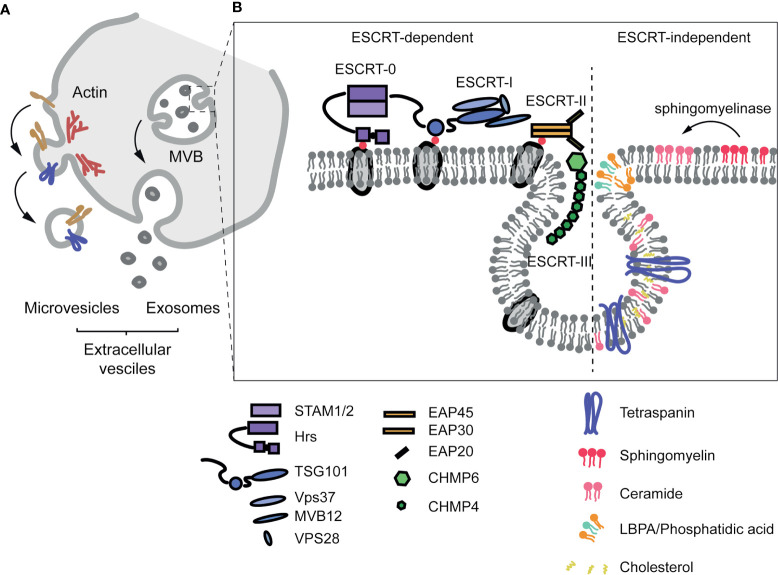
Extracellular vesicle formation. **(A)** Extracellular vesicles are classified according to their origin. Microvesicles are formed from the PM in a process called ectocytosis that depends on the ESCRT machinery and the actin cytoskeleton. Exosomes are derived from the budding process on the membrane of the endosome/multivesicular body, and released to the extracellular space upon fusion of the endosome with the PM. **(B)** Diagram of the structure of the ESCRT machinery and of ESCRT-independent mechanisms implicated in exosome formation.

On the other hand, MVs are derived from scission of small plasma membrane-derived vesicles (110). This process—termed ectocytosis—shares many similar steps to exosome formation. The ESCRT machinery, as well as cytoskeletal elements and their regulators, such as RHO family of GTPases and ROCK, are important for the formation of MVs together with other membrane-associated proteins, including tetraspanins and membrane cargos (124) (**Figure 2**).

Virtually every cell type can release EVs, and these structures are abundant in the extracellular space and body fluids such as plasma, urine and saliva. A broad spectrum of cargoes (e.g. nucleic acids, proteins or signalling molecules) can be sorted onto these vesicles and subsequently exported and transferred to target cells. Many cargoes have been related to the modulation of the biology of acceptor cells in multiple physiologic and pathologic scenarios.

Immune responses are exquisitely regulated to ensure defence from external pathogens or physicochemical insults as well as internal alterations such as tumor cell growth, while avoiding damage of the self. EVs are crucial in the intricate cell-cell communication involved. EVs are frequently described as pro-inflammatory mediators and participate in the propagation of inflammatory signals during infections and chronic inflammatory diseases among components of innate immunity. Mechanistically, several cargoes such as cytokines, receptors and microRNAs can modulate the activation state and function of macrophages, neutrophilic granulocytes and natural killer (NK) cells (125). EVs also participate of several steps of acquired immunity and antigen presentation. Antigen presenting cells (APCs), including B lymphocytes, dendritic cells (DCs) and macrophages can release major histocompatibility complex II (MHC-II) through exosomes enabling antigen presentation to CD4^+^ T lymphocytes at distance (126). EVs released by tumor cells or several pathogens can constitute a relevant source of antigens for APCs for their processing and presentation to CD4^+^ T lymphocytes (127, 128). EVs also actively participate of the immune synapse between lymphocytes and APCs, and lymphocytes specifically relocalize multivesicular bodies (MVBs) towards the contact site, leading to a localized increase in exosome secretion and unidirectional transfer of microRNAs that modulate downstream responses. Highlighting the key role of EV communication in this process, inhibition of exosomes formation/secretion dysregulates gene expression in APCs (129) and reduces antibody production in activated B-cells (130, 131).

Immune cell-derived EVs are also involved in other inflammatory processes such as tissue fibrosis, where an increase in ECM deposition has been described to impact cell behaviour, including cell proliferation, migration and differentiation, and subsequently participating in the development of several pathologies. A prominent EVs profibrotic cargo is interleukin-1β (IL-1β) (132), which is released by DCs upon binding of ATP to P2X purinoceptor 7 (P2X7R) (133) and can act on several IL-1β) receptor-expressing cell types (134, 135). IL-1β can, in turn, induce vesicular secretion of interleukin-6 (IL-6) in mast cells, amplifying inflammation (136). Other ligands that induce fibrosis such as TGF-β or TNFα have also been described as EVs cargoes.

EVs-dependent secretion and inter-tissue communication is also involved in vascular physiopathology (137–140). EVs-mediated communication can be involved in either AS progression or lesion prevention. Krüppel-like factor 2 (KLF2)-expressing endothelial cells (ECs) (an atheroprotective hallmark) can load miR-143/145 in exosomes to control smooth muscle cell (SMC) activation and reduce AS lesion formation (141). In contrast, proinflammatory cues on ECs repress the presence of Ten-eleven translocation 2 (TET2) dioxygenase in exosomes, promoting plaque formation (142). SMCs can influence back endothelial function through EVs: SMC-derived E cargo miR-155 increases endothelial permeability (143). EVs also play a role in the development of an inflammatory environment in the progressing atherosclerotic plaque (144–146). EVs may also directly contribute to subendothelial matrix remodeling and lesion progression, either through recently discovered ECM deposition (see below), as well as sphingomyelin phosphodiesterase 3 (SMPD3)-dependent calcification (147).

Myocardial injury engages mechanisms to repair and maintain cardiac function, including cardiac fibrosis by activation of resident fibroblasts through TGF-β, EDN-1, PDGF, CCN2 and AGTII ligands, which can be released through EVs derived from cardiomyocytes and ECs. Reflecting a role in events after myocardial injury, miRNAs cargo signatures on EVs (including miR-1, -208, -214) (148, 149) emerge as good biomarkers of myocardial infarction detection and prognosis from plasma samples.

Tumor cells (TCs) usually secrete large amounts of EVs, which can influence different aspects of tumor progression and behaviour, including tumour-associated fibroblast activation, angiogenesis, immunomodulation, matrix remodeling or the establishment of pre-metastatic niches. TC populations are heterogeneous (150–152). TCs communicate inside the tumor and can transfer part of their unique characteristics to other surrounding cancer cells. For example, tumour-derived EVs can modulate local growth *via* autocrine transfer of mutant KRAS proto-oncogene to wild type KRAS-expressing colon cancer cells (153). Similarly, glioblastoma microvesicles transport specific RNAs that promote neighbour proliferation (154). EVs can also transmit their capacity to adapt to the characteristic tumor stresses such as hypoxia, changes in pH and nutrient deprivation (155).

Tumor angiogenesis and abnormal vascularization determines its behaviour and response to therapy (156, 157). Many pro-angiogenic factors are tumoral EV cargoes, such as the vascular endothelial growth factor (VEGF), platelet-derived growth factor (PDGF), TGF-b, TNF-a or fibroblast growth factor (FGF) (158). Tumour-derived exosomes can also induce vascular permeability in distant organs in breast, melanoma and colorectal cancers (159–161).

Antitumor immunity and its suppression by tumors are another major focus of research and therapeutic intervention, and EVs also play a role in this process. DCs induce T-cell and NK cell activation in an EV-dependent manner to mount an antitumor response (126, 162–164). As the tumor progresses, TCs deploy mechanisms such as attenuation of NK cell cytotoxicity (block of NKG2D pathway), reduction of T-cell-mediated killing or activation of myeloid-derived suppressor cells (TC-derived EVs can contain PGE2, TGF-b and HSP72) (127).

Under physiological conditions, fibroblasts are in a quiescent state. Upon tissular damage, they can enter an activated state, whereby a “secretory phenotype”—to produce both paracrine signals and new ECM components—and contractile activity—for the biomechanical remodeling of tissue—are acquired. Dysregulated persistent activation is a hallmark of tumour-associated fibroblasts (TAFs) (165) and other pathological conditions coursing with fibrosis and desmoplasia. Tumour-derived EVs can induce fibroblast activation (166), by virtue of microRNA cargo subsets modulating motility, collagen contraction or proliferation (167). TC-derived exosomes can also induce secretion of specific ECM components, such as FN (168, 169), as well as ECM remodeling enzymes (170, 171).

Evidence suggests that EVs can actively participate of ECM sculpting (172, 173), through ECM remodeling cargoes such as MMPs (174) or lysyl oxidases (175, 176). Active MMPs such as MMP-1, -13, -2, -3 or -14 are detected on the surface of EVs derived from several tumor cell types. Moreover, ADAMs family (regulators of cell adhesion and migration) components and more specifically the two most notorious members of this family (ADAM10 and ADAM17) have been described as EVs cargoes (177, 178).

## EVs as ECM Carriers: Implications IN ECM Secretion

Recent studies support that some ECM components are EVs cargoes themselves, implying that trafficking and export mechanisms could coexist with canonical secretion pathways, modulating ECM composition and architecture and subsequently impacting on cell behaviour. Additionally, EV function could also be linked to ECM remodelling in the sense that ECM fiber components might influence the retention of EVs at specific regions through discrete subsets of receptors in their surface (168), therefore contributing to their selectivity for cell type targeting and favouring a specific evolving composition and architecture of the ECM during its remodeling.

Early observations hinting at the involvement of EVs in matrix secretion and deposition described “matrix vesicles” (179, 180), as a relevant mechanism for osteoblast-mediated primary bone mineralization (181, 182). Secreted matrix vesicles initiate the nucleation of calcium phosphate crystals by an influx of Ca^2+^ and PO_4_^3-^ through their membrane transporters and the action of several intraluminal enzymes such as tissue-nonspecific alkaline phosphatase (TNSALP), ectonucleotide pyrophosphatase/phosphodiesterase 1 (ENPP1) or phosphoethanolamine/phosphocholine phosphatase 1 (PHOSPHO1) (182). Interestingly, a role for matrix vesicles has been also described in vascular SMC-driven calcification during AS progression (147).

**Figure 3:**
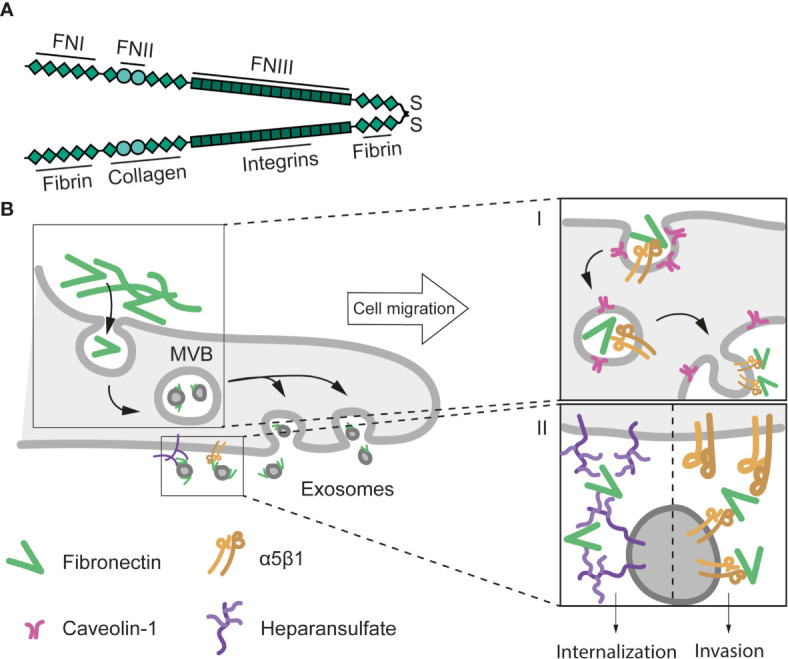
Structure and exosomal secretion of FN. **(A)** Structure of a FN dimer stabilised by a di-sulphide bond. Basic domains (FNI, II, and II) and the main binding sites to other ECM proteins and receptors are depicted. **(B)** Exosome-mediated FN secretion and cell migration. Caveolin-1-dependent β1 integrin endocytosis is implicated in the internalization of extracellular FN. Upon endocytosis, FN is transported to endosomes, where exosomes are formed (stage I) (189). Exosomal FN is then released at invadopodia, and induces cell migration and invasion through its internalization (138) or *via* activation of integrin-mediated pathways (192) (stage II).

Our understanding of the implication of EVs in the secretion and deposition of specific ECM components has since considerably lagged. Recent studies have shown that ECM proteins are exported and deposited by EVs (183, 184) (www.vesiclepedia.org, www.exocarta.org) and animal models in which exosome production has been abrogated through disruption of neutral sphingomyelinase (NSMase) activities show marked alterations in ECM deposition and architecture (185, 186). FN is a prominent ECM cargo in EVs from different cell types, and the blockade of exosome secretion partially alters, although does not completely impair, FN fiber deposit (108). Other groups have recently described that FN is transported by EVs. FN accumulates at the surface of exosomes through its binding to heparan-sulfate (187, 188) and that upon beta1 integrin endocytosis (189), FN can be redeposited from the endosomal compartment at the basal cell surface in epithelial cells (cortactin-dependent) (190) and in epicardial cells (mediated by Bves and NDRG4) (191). Moreover, Weaver and colleagues suggested that exosome secretion plays a key role in autocrine deposition of FN at the leading edge of the cells: Golgi secreted FN would be in an inactive form previous to its assembly at the cell surface (38), exosomal FN, presumably sourced from the endosomal compartment (109, 189, 191) would constitute a rapid alternative pathway for competent adhesive substrate deposition (**Figure 3**).

FN-containing EVs have been associated with tumor progression. Certain features of tumor cells can be altered by the presence of FN-positive EVs in the media. Weaver and coworkers have characterized that exosome secretion in invadopodia is essential for FN resecretion, and regulates cell adhesion, directional motility and invasion in tumor cells (109, 192, 193). Invasiveness of fibroblasts is positively regulated by FN-positive EVs treatment (194). Exosomal FN can modulate other functional programmes such as proliferation (195), signal transduction (138, 196), endocytosis (197) or cell survival (198). Finally, exosomal FN can modulate tumor immunity. Secretion of FN-containing EVs can be induced by tumour-associated leukocytes (199), but these FN pools can also induce pro-inflammatory IL-1β) production by macrophages (200). FN interacts with acceptor cells through plasma membrane heparansulfate and α5 integrin receptor (109, 187, 200). However, the mechanism of action of exosomal FN seems to require its internalization (138). This apparent discrepancy may indicate that exosomal ECM could be activating several pathways depending on the mechanism by which they interact with acceptor cells (**Table 1**).

**Table 1:** Literature contributing evidence of FN as an EV secreted cargo. EVs origin: cell type/tissue from which EVs containing TnC were detected; WB: western blotting.

FN					
EVs origin	Target cell	Detection approach	Result		Ref.
Myeloma RPMI-8226 and CAG)	Human bone marrow stroma (HS-5), Human umbilical vein endothelial cells	WB and light microscopy		Exosome-cell interaction and internalization	(187)
				Myeloma tumour growth and progression (p38 and pERK activation)	
				Increased endothelial cell invasion	
Fibrosarcoma (HT1080)	Fibrosarcoma (HT1080)	WB and sucrose gradient		Increased motility	(192)
HIV-1 infected dendritic cells	T-lymphocyte	WB		Viral trans-infection	(201)
				Increased IFN-γ, TNF-α, IL-1β and RANTES	
				Activation of p38/Stat pathways	
Human trabecular meshwork cells	N/A	WB		Dexamethasone reduces exosomal FN levels	(188)
Fibrosarcoma (HT1080)	Fibrosarcoma (HT1080)	WB and sucrose gradient		Tumour cell migration	(109)
Transplantation patient serum	N/A	WB		Allograft rejection biomarker	(202)
Human trophoblast	Macrophage	WB		Increased IL-1β production	(200)
Mesenchymal stem cells	Bone marrow (SH-SY5Y)	WB and Proteomics		Increased mitosis and growth factor secretion	(195)
Endothelial cells	Hepatic stellate cell	WB and electron microscopy		Increased AKT phosphorylation	(138)
				Increased cell migration	
Tumour-associated leukocytes	Breast cancer (AT-3)	WB and FACS		Increased exosomal FN	(199)
	Colon cancer (4T1, CT26)			Increased tumour cell invasion	
Fibroblast (IMR90)	Fibroblast (IMR90)	Proteomics, FACS		Fibroblast invasion	(194)
Primary melanocyte	Primary melanocyte	WB, proteomics, light microscopy		Increased melanocyte survival after UVB radiation	(198)
Microvascular endothelial cells	Oligodendrocyte precursor cell (OPC)	Proteomics, enzyme-linked immunosorbent assay		OPCs survival and proliferation	(197)

## EV Secretion: An Integral Aspect of TnC Biological Roles

**Figure 4:**
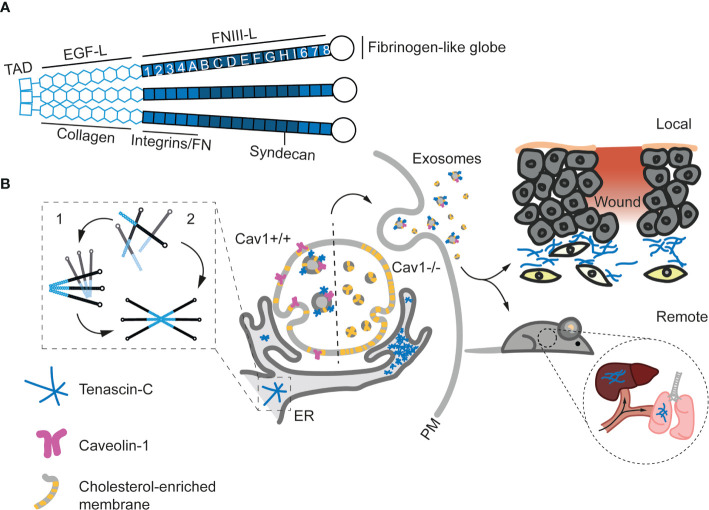
Structure and exosomal secretion of TnC. **(A)** Structure of a trimer of TnC. Tenascin monomers bind *via* the tenascin assembly domain (TAD) located at the N-terminus. Basic domains (EGF-Like and FNIII-Like) and the main binding sites to other ECM proteins and receptors are depicted. **(B)** Models for TnC biosynthesis. In model 1, hexabrachions are formed in a very rapid co-translational process where six monomers are simultaneously assembled. In model 2, the hexabrachion assembly take place in two steps. First, monomers form an intermediary trimer through α-helical coiled-coil interactions in the TAD. Subsequently, two trimers assemble in a hexamer that is stabilized by di-sulphide bonds. **(C)** Exosome-mediated TnC secretion. After biosynthesis in the ER, TnC is transported to multivesicular bodies (MVBs) in a Cav1 dependent manner (Cav1^+/+^). The absence of Cav1 (Cav1^-/-^) increases the levels of cholesterol at MVBs and alters exosome formation, preventing the sorting of TnC onto exosomes and leading to the accumulation of TnC in the ER. Upon secretion, exosomal TnC can be locally deposited, or modulate the behavior of surrounding cells. On the other hand, exosomes can eventually reach the blood stream and generate new TnC nucleation points at distant organs and tissues (108).

Recent studies demonstrate that exosome secretion is strictly required for appropriate extracellular TnC deposition by both tumor cells and different fibroblast types (108, 122) (**Figure 4**). Circulating exosomes from cancer patients frequently carry TnC (24), and several cancer cell types secrete TnC in EVs *in vitro* (183, 184) (www.microvesicle.org, www.exocarta.org). Disruption of exosome secretion by pharmacological inhibition or RNAi-mediated depletion of NSMase 2 led to accumulation of TnC at the ER and decreased extracellular TnC fibre formation. These studies excluded internalization of extracellular TnC and established that exosome-secreted TnC is synthesized *de novo*. Mechanistically, caveolin-1 [Cav1; a pivotal regulator of membrane organization, mechanoadaptation, ECM remodeling and cholesterol efflux (16, 203–205)] is strictly required for the appropriate biogenesis of exosome subpopulations of different sizes, and the sorting onto them of specific ECM components, through the control of cholesterol content in endosomal compartments. Interestingly, this effect varies across ECM exosome cargoes, suggesting that the extent of dependency on different secretion routes may be specific for each ECM component; for example, in contrast with TnC, FN deposition is only partially decreased upon disruption of exososomal secretion. Cav1 deficiency, exogenous cholesterol loading or pharmalogical inhibition of cholesterol trafficking from endosomes all markedly impaired exosomal secretion of TnC. Cholesterol homeostasis emerges as an as yet poorly understood mechanism by which membrane trafficking and metabolism potentially feed onto functions allocated at the endosomal compartment, including cell signalling regulation (206, 207) and exosome secretion (108).

The involvement of Cav1 as a central regulator of this process is not trivial. Cav1 is a central node simultaneously regulating the transduction of information on ECM composition and physical properties (204), and the coordinated remodeling of both aspects (16, 108, 208). This reciprocal crosstalk [first discussed by Bissell and Hall as stromal dynamic reciprocity (209)] is key to understand both physiological and pathological processes pertaining different tissues. Furthermore, collagens are not a class of ECM components correlating with TnC in their Cav1-dependent sorting onto exosomes; in fact, Cav1 might regulate oppositely COPII-dependent deposition of collagen, and exosome-mediated secretion of other ECM components (210). It remains to be studied whether other components of caveolae such as PTRF—which does appear to modulate exosome-mediated secretion (211)—also regulate the sorting of ECM components to exosomes. Cav1-dependent regulation of tissue architecture and cell function is relevant for several conditions in which TnC has a prominent role, such as tumor progression or cardiovascular remodeling (16, 212, 213). An additional standing question is whether Cav1 expression (both during exosome biogenesis as well as at destination) may determine the specificity of exosome-mediated communication, given the prominent role integrins appear to have in this process (168).

**Figure 5:**
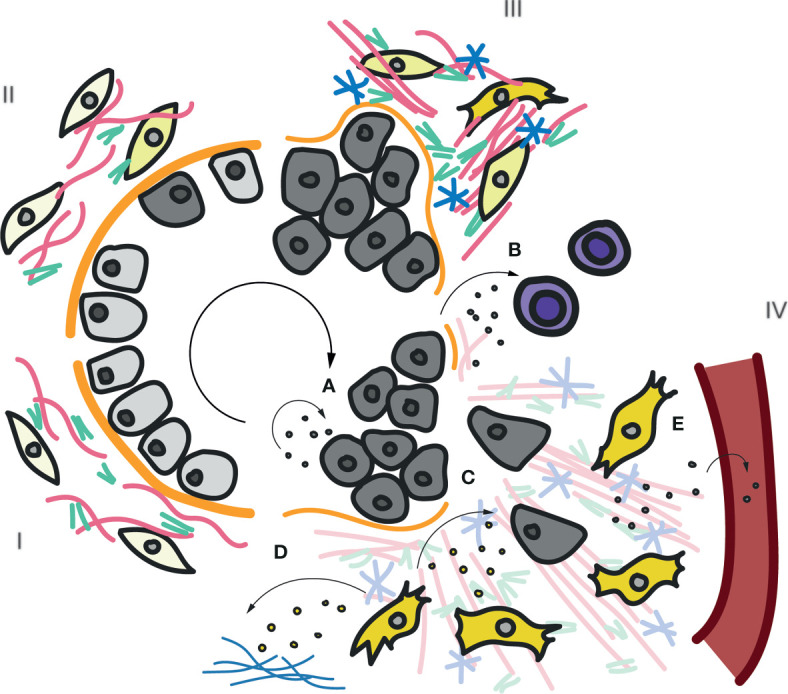
Roles of exosomal TnC in cancer progression and immunomodulation. Scheme of the main stages (I-IV) in carcinoma tumor progression. In stage I a normal epithelium is shown, composed by epithelial cells located on a basal membrane. Underneath, the interstitial matrix deposited by stromal cells provides support. Insults promote transformation of epithelial cells onto tumoral cells, which lose polarity and adhesion (Stage II). In stage III, continuously activated fibroblasts increase the production and secretion of ECM, including collagens, FN and TnC. Tumor cells start invading neighbouring tissues and degrading the basal membrane. Finally, in stage IV, a highly remodelled ECM favors tumor cell migration through the interstitial space towards blood and lymphatic vessels, to metastasize. The previously described roles of exosomal TnC in tumor progression are depicted **(A–E)**. **(A)** Paracrine/autocrine secretion of TnC-loaded exosomes induce tumor cell proliferation and invasion (122). **(B)** Exosomal TnC derived from brain tumour-initiating cells suppresses mTOR activity and T-cell activity (219)(Mirzaei et al). Activated fibroblasts can also secrete exosomes carrying TnC that can **(C)** modulate tumor cells and/or deposit new TnC matrix (108) **(D)**. Finally, TnC-positive exosomes can be released into the bloodstream to deposit TnC at distant organs **(E)**. An increase in TnC in plasma has been proposed as poor prognosis marker in many cancers and inflammatory diseases (24).

Exosome secretion appears to account for the major share (if not the totality) of TnC extracellular release and deposition (108); thus, virtually all biological/physiopathological roles of TnC should be framed by the specific features of exosomal communication. Exosomes enable the transport of cargoes across interorgan distances, and TnC-containing exosomes can nucleate ECM beds in different organs of TnCKO mice such as liver and lungs upon intravenous injection (108); these observations suggest that exosomal deposit of TnC and associated ECM components contributes significantly to pre-metastatic niche formation (169). These pools of exosomal TnC are fully functional and apart from fostering ECM fiber nucleation, efficiently induce proinflammatory states and features compatible with EMT in breast cancer cells in 2D and 3D culture models (108, 122). Exosomal TnC levels also correlate with invasiveness in pancreatic ductal adenocarcinoma (184, 214), and induce invasion through WNT/β-catenin signaling, a crucial pathway in EMT modulation, and activation of the NF/κB pathway (214). Exosomes have also recently emerged as efficient platforms for immunomodulation in the tumor microenvironment and other tissue contexts (131, 215); it is likely that the prominent roles TnC has as a regulator of immune cell function (see first section) are exerted at least in part through exosomes. Interestingly, exosomes released by SARS-CoV2-infected cells are significantly enriched in TnC and could promote the propagation of inflammation to distant sites (216). Serum TnC levels have been explored as diagnostic/prognostic markers in different pathologies (24), but whether all circulating TnC is exclusively trafficked through EVs is yet to be determined. Other examples of paracrine secretion of TnC in exosomes include osteoblasts (217), airway epithelial cells (218) and several tumor cells (183, 184), where exosomal TnC has been associated to alterations of pre-existing ECM, impacting collagen and alkaline phosphatase activity. Yong and co-workers also described that brain tumour-initiating cells can secrete TnC in exosomes and suppress T-cell activation, enabling tumor progression and metastasis through the modulation of antitumor immunity (219). Mechanistically, TnC could inhibit T-cell activation and proliferation through the well-established TnC receptors α5β1 and ανβ6 integrins, reducing mTOR signaling (**Figure 5** and **Table 2**).

**Table 2:** Literature contributing evidence of TnC as an EV secreted cargo. EVs origin: cell type/tissue from which EVs containing TnC were detected; WB: western blotting.

TnC					
EVs origin	Target cell	Detection approach	Result		Ref.
Fibroblast	Breast cancer (MDA-MB-468)	WB, sucrose gradient and proteomics	Matrix deposition in 2D, 3D and *in vivo*		(108)
			increased migration and invasion		
Breast cancer (MDA-MB-231)	Breast cancer (MDA-MB-231, T47-D)	WB, proteomics	increased migration and invasion		(122)
Brain tumor-initiating cells	T-lymphocyte	WB	Inhibition of mTOR signalling and inhibition of T-cell proliferation, activation and cytokine secretion		(219)
Glioblastoma patients					
Osteoblast-like cells (SaOS2)	N/A	WB	Bone mineralization		(217)
Pancreatic cancer (PC-1, PC-1.0, AsPC-1, Capan-2)	Pancreatic cancer	WB	Increased migration and invasion		(214)
			Increased proliferation through activation of the NF/kB		
Metastatic colorectal cancer (SW480, SW620)	N/A	Proteomics	Increased exosomal TnC in metastatic cell lines		(183)
Pancreatic ductal adenocarcinoma patients (pancreatic duct fluid)	N/A	Proteomics	Increased exosomal TnC correlates with stromal TnC matrix		(184)

Additionally, it may be considered that TnC fibers at a given ECM niche could act as efficient receptors for the homing of exosome subsets exposing TnC-binding receptors, a mechanism that may contribute to ECM remodeling and its coordination with cell modulation. Finally, the consideration of features derived from exosomal secretion might be highly relevant for biomedical applications aiming at tissue repair and regeneration: exosomes would potentially enable for accurate “dosage” and target specificity (220), and might hold the key for leveraging on the tissue remodeling and repair activities of TnC (221) through very controlled time frames, bypassing uncontrolled chronic inflammation states.

## Concluding Remarks and Perspectives

The characterization of mechanisms driving ECM deposit and of antifibrotic agents (72, 222, 223) aiming at intervening or preventing diseases such as chronic hepatitis (224), kidney diseases (225), systemic sclerosis, pulmonary fibrosis (226, 227) or cancer and tumor progression (228) has been intensive. Throughout the past decade, the study of EV-associated ECM components has expanded our understanding of ECM biology. EVs have been suggested as integral components of stromal environments (172, 173), and enable the impact of ECM-secreting cell populations on distant organismal locations. These insights have opened several key questions. We do not know whether EV-mediated transport of certain ECM components specifies their function at destiny. Mechanistically, we have a very limited understanding as to how ECM components are routed for sorting onto exosomes, instead of being targeted for degradation at the endosomal compartment; whether and how cells use different potential mechanisms for the secretion of a given ECM component; and how these processes are integrated with the complex reciprocal regulation established between ECM and stromal cells. Finally, the principles by which target cell specificity (168) correlates with this ECM secretion activity remain unexplored. The potential interplay of EV-carried TnC with other cargoes regarding their impact on target cells is also a key question. Given the potential of EV-trafficked TnC levels as serum diagnosis/prognosis biomarkers, and the ability of EVs to nucleate novel ECM niches at specific organs, the biology of exosomal TnC secretion holds the promise to explore potential novel theranostic applications.

## Author Contributions

LA-A, MS-Á, and MP conceived and wrote the article. LA-A created all infographics, and led bibliographical revision with support from MS-Á. MP coordinated the review. All authors contributed to the article and approved the submitted version.

## Funding

The authors acknowledge the grant support to MP from the Spanish Ministerio de Ciencia e Innovación (CSD2009-0016, SAF2014-51876-R, SAF2017-83130-R, BFU2016-81912-REDC, and IGP-SO-MINSEV1512-07-2016), Fundació La Marató de TV3 (674/C/2013 and 201936-30-31), Worldwide Cancer Research Foundation (# 15-0404), Asociación Española Contra el Cáncer (PROYE20089DELP), and Fondo Europeo de Desarrollo Regional *”Una manera de hacer Europa”*. MP received funding from the European Union Horizon 2020 research and innovation program under Marie Sklodowska-Curie grant agreement no. 641639, and is member of the Tec4Bio consortium (ref. S2018/NMT4443; Actividades de I+D entre Grupos de Investigación en Tecnologías, Comunidad Autónoma de Madrid/FEDER, Spain). LA-A was supported by a Ministerio de Ciencia, Innovación y Universidades predoctoral fellowship associated with the Severo Ochoa Excellence program (ref. SVP-2013-06789). The Centro Nacional de Investigaciones Cardiovasculares Carlos III is supported by the Ministerio de Ciencia e Innovación and the Pro CNIC Foundation and is a Severo Ochoa Center of Excellence (SEV-2015-0505).

## Conflict of Interest

The authors declare that the research was conducted in the absence of any commercial or financial relationships that could be construed as a potential conflict of interest.

## References

[1] Hynes RO. The Evolution of Metazoan Extracellular Matrix. J Cell Biol (2012) 196:671–9. 10.1083/jcb.201109041 PMC3308698 22431747

[2] Ozbek S Balasubramanian PG Chiquet-Ehrismann R Tucker RP Adams JC. The Evolution of Extracellular Matrix. Mol Biol Cell (2010) 21:4300–5. 10.1091/mbc.e10-03-0251 PMC3002383 21160071

[3] Frantz C Stewart KM Weaver VM. The Extracellular Matrix At a Glance. J Cell Sci (2010) 123:4195–200. 10.1242/jcs.023820 PMC2995612 21123617

[4] Cox TR Erler JT. Remodeling and Homeostasis of the Extracellular Matrix: Implications for Fibrotic Diseases and Cancer. Dis Model Mech (2011) 4:165–78. 10.1242/dmm.004077 PMC3046088 21324931

[5] Egeblad M Rasch MG Weaver VM. Dynamic Interplay Between the Collagen Scaffold and Tumor Evolution. Curr Opin Cell Biol (2010) 22:697–706. 10.1016/j.ceb.2010.08.015 20822891 PMC2948601

[6] Kass L Erler JT Dembo M Weaver VM. Mammary Epithelial Cell: Influence of Extracellular Matrix Composition and Organization During Development and Tumorigenesis. Int J Biochem Cell Biol (2007) 39:1987–94. 10.1016/j.biocel.2007.06.025 PMC2658720 17719831

[7] Bonnans C Chou J Werb Z. Remodelling the Extracellular Matrix in Development and Disease. Nat Rev Mol Cell Biol (2014) 15:786–801. 10.1038/nrm3904 25415508 PMC4316204

[8] Mott JD Werb Z. Regulation of Matrix Biology by Matrix Metalloproteinases. Curr Opin Cell Biol (2004) 16:558–64. 10.1016/j.ceb.2004.07.010 PMC2775446 15363807

[9] Arpino V Brock M Gill SE. The Role of Timps in Regulation of Extracellular Matrix Proteolysis. Matrix Biol (2015) 44–46:247–54. 10.1016/j.matbio.2015.03.005 25805621

[10] Trackman PC. Functional Importance of Lysyl Oxidase Family Propeptide Regions. J Cell Commun Signal (2018) 12:45–53. 10.1007/s12079-017-0424-4 29086201 PMC5842187

[11] Alexander J Cukierman E. Stromal Dynamic Reciprocity in Cancer: Intricacies of Fibroblastic-ECM Interactions. Curr Opin Cell Biol (2016) 42:80–93. 10.1016/j.ceb.2016.05.002 27214794 PMC5064819

[12] Hynes RO. The Extracellular Matrix: Not Just Pretty Fibrils. Science (80-) (2009) 326:1216–9. 10.1126/science.1176009 PMC3536535 19965464

[13] Pickup MW Mouw JK Weaver VM. The Extracellular Matrix Modulates the Hallmarks of Cancer. EMBO Rep (2014) 15:1243–53. 10.15252/embr.201439246 PMC4264927 25381661

[14] Calvo F Ege N Grande-Garcia A Hooper S Jenkins RP Chaudhry SI. Mechanotransduction and YAP-Dependent Matrix Remodelling is Required for the Generation and Maintenance of Cancer-Associated Fibroblasts. Nat Cell Biol (2013) 15:637–46. 10.1038/ncb2756 PMC3836234 23708000

[15] Mohammadi H Sahai E. Mechanisms and Impact of Altered Tumour Mechanics. Nat Cell Biol (2018) 20:766–74. 10.1038/s41556-018-0131-2 29950570

[16] Goetz JG Minguet S Navarro-Lérida I Lazcano JJ Samaniego R Calvo E. Biomechanical Remodeling of the Microenvironment by Stromal Caveolin-1 Favors Tumor Invasion and Metastasis. Cell (2011) 146(1):148–63. 10.1016/j.cell.2011.05.040 PMC3244213 21729786

[17] Valcourt U Alcaraz LB Exposito JY Lethias C Bartholin L. Tenascin-X: Beyond the Architectural Function. Cell Adh Migr (2015) 9:154–65. 10.4161/19336918.2014.994893 PMC4422802 25793578

[18] Marini JC Forlino A Cabral WA Barnes AM San Antonio JD Milgrom S. Consortium for Osteogenesis Imperfecta Mutations in the Helical Domain of Type I Collagen: Regions Rich in Lethal Mutations Align With Collagen Binding Sites for Integrins and Proteoglycans. Hum Mutat (2007) 28:209–21. 10.1002/humu.20429 PMC4144349 17078022

[19] Pober BR. Williams-Beuren Syndrome. N Engl J Med (2010) 362:239–52. 10.1056/NEJMra0903074 20089974

[20] Jarvelainen H Sainio A Koulu M Wight TN Penttinen R. Extracellular Matrix Molecules: Potential Targets in Pharmacotherapy. Pharmacol Rev (2009) 61:198–223. 10.1124/pr.109.001289 19549927 PMC2830117

[21] Wynn TA Ramalingam TR. Mechanisms of Fibrosis: Therapeutic Translation for Fibrotic Disease. Nat Med (2012) 18:1028–40. 10.1038/nm.2807 PMC3405917 22772564

[22] Jones FS Jones PL. The Tenascin Family of ECM Glycoproteins: Structure, Function, and Regulation During Embryonic Development and Tissue Remodeling. Dev Dyn (2000) 218:235–59. 10.1002/(SICI)1097-0177(200006)218:2<235::AID-DVDY2>3.0.CO;2-G 10842355

[23] Huang W Chiquet-Ehrismann R Moyano JV Garcia-Pardo A Orend G. Interference of Tenascin-C With Syndecan-4 Binding to Fibronectin Blocks Cell Adhesion and Stimulates Tumor Cell Proliferation. Cancer Res (2001) 61:8586–94. 11731446

[24] Giblin SP Midwood KS. Tenascin-C: Form Versus Function. Cell Adh Migr (2015) 9:48–82. 10.4161/19336918.2014.987587 25482829 PMC4422809

[25] Midwood KS Chiquet M Tucker RP Orend G. Tenascin-C At a Glance. J Cell Sci (2016) 129:4321–7. 10.1242/jcs.190546 27875272

[26] Midwood KS Hussenet T Langlois B Orend G. Advances in Tenascin-C Biology. Cell Mol Life Sci (2011) 68:3175–99. 10.1007/s00018-011-0783-6 PMC3173650 21818551

[27] Tucker RP Chiquet-Ehrismann R. Tenascin-C: its Functions as an Integrin Ligand. Int J Biochem Cell Biol (2015) 65:165–8. 10.1016/j.biocel.2015.06.003 26055518

[28] Yoshida T Akatsuka T Imanaka-Yoshida K. Tenascin-C and Integrins in Cancer. Cell Adh Migr (2015) 9:96–104. 10.1080/19336918.2015.1008332 25793576 PMC4422796

[29] Wiemann S Reinhard J Faissner A. Immunomodulatory Role of the Extracellular Matrix Protein Tenascin-C in Neuroinflammation. Biochem Soc Trans (2019) 47:1651–60. 10.1042/BST20190081 31845742

[30] Imanaka-Yoshida K. Inflammation in Myocardial Disease: From Myocarditis to Dilated Cardiomyopathy. Pathol Int (2020) 70(1):1–11. 10.1111/pin.12868 31691489

[31] Udalova IA Ruhmann M Thomson SJ Midwood KS. Expression and Immune Function of Tenascin-C. Crit Rev Immunol (2011) 31:115–45. 10.1615/CritRevImmunol.v31.i2.30 21542790

[32] Unlu G Levic DS Melville DB Knapik EW. Trafficking Mechanisms of Extracellular Matrix Macromolecules: Insights From Vertebrate Development and Human Diseases. Int J Biochem Cell Biol (2014) 47:57–67. 10.1016/j.biocel.2013.11.005 24333299 PMC3915713

[33] Chiquet-Ehrismann R. What Distinguishes Tenascin From Fibronectin? FASEB J (1990) 4:2598–604. 10.1096/fasebj.4.9.1693347 1693347

[34] Adams JC Chiquet-Ehrismann R Tucker RP. The Evolution of Tenascins and Fibronectin. Cell Adh Migr (2015) 9:22–33. 10.4161/19336918.2014.970030 25482621 PMC4422808

[35] Schafer M Werner S. Cancer as an Overhealing Wound: An Old Hypothesis Revisited. Nat Rev Mol Cell Biol (2008) 9:628–38. 10.1038/nrm2455 18628784

[36] Hanahan D Weinberg RA. Hallmarks of Cancer: The Next Generation. Cell (2011) 144:646–74. 10.1016/j.cell.2011.02.013 21376230

[37] Quail DF Joyce JA. Microenvironmental Regulation of Tumor Progression and Metastasis. Nat Med (2013) 19:1423–37. 10.1038/nm.3394 PMC3954707 24202395

[38] Singh P Carraher C Schwarzbauer JE. Assembly of Fibronectin Extracellular Matrix. Annu Rev Cell Dev Biol (2010) 26:397–419. 10.1146/annurev-cellbio-100109-104020 20690820 PMC3628685

[39] Pankov R Yamada KM. Fibronectin At a Glance. J Cell Sci (2002) 115:3861–3. 10.1242/jcs.00059 12244123

[40] Katoh D Nagaharu K Shimojo N Hanamura N Yamashita M Kozuka Y. Binding of Alphavbeta1 and Alphavbeta6 Integrins to Tenascin-C Induces Epithelial-Mesenchymal Transition-Like Change of Breast Cancer Cells. Oncogenesis (2013) 2:e65. 10.1038/oncsis.2013.27 23958855 PMC3759126

[41] Bates RC Bellovin DI Brown C Maynard E Wu B Kawakatsu H. Transcriptional Activation of Integrin Beta6 During the Epithelial-Mesenchymal Transition Defines a Novel Prognostic Indicator of Aggressive Colon Carcinoma. J Clin Invest (2005) 115:339–47. 10.1172/JCI200523183 PMC544606 15668738

[42] Ramos DM Dang D Sadler S. The Role of the Integrin Alpha V Beta6 in Regulating the Epithelial to Mesenchymal Transition in Oral Cancer. Anticancer Res (2009) 29:125–30. 19331141

[43] Nagaharu K Zhang X Yoshida T Katoh D Hanamura N Kozuka Y. Tenascin C Induces Epithelial-Mesenchymal Transition-Like Change Accompanied by SRC Activation and Focal Adhesion Kinase Phosphorylation in Human Breast Cancer Cells. Am J Pathol (2011) 178:754–63. 10.1016/j.ajpath.2010.10.015 PMC3069868 21281808

[44] Yokosaki Y Monis H Chen J Sheppard D. Differential Effects of the Integrins Alpha9beta1, Alphavbeta3, and Alphavbeta6 on Cell Proliferative Responses to Tenascin. Roles of the Beta Subunit Extracellular and Cytoplasmic Domains. J Biol Chem (1996) 271:24144–50. 10.1074/jbc.271.39.24144 8798654

[45] Saupe F Schwenzer A Jia Y Gasser I Spenle C Langlois B. Tenascin-C Downregulates Wnt Inhibitor Dickkopf-1, Promoting Tumorigenesis in a Neuroendocrine Tumor Model. Cell Rep (2013) 5:482–92. 10.1016/j.celrep.2013.09.014 24139798

[46] Lowy CM Oskarsson T. Tenascin C in Metastasis: A View From the Invasive Front. Cell Adh Migr (2015) 9:112–24. 10.1080/19336918.2015.1008331 PMC4422797 25738825

[47] Shao H Kirkwood JM Wells A. Tenascin-C Signaling in Melanoma. Cell Adh Migr (2015) 9:125–30. 10.4161/19336918.2014.972781 PMC4422811 25482624

[48] Kimura T Tajiri K Sato A Sakai S Wang Z Yoshida T. Tenascin-C Accelerates Adverse Ventricular Remodelling After Myocardial Infarction by Modulating Macrophage Polarization. Cardiovasc Res (2019) 115(3):614–24. 10.1093/cvr/cvy244 30295707

[49] Manrique-Castano D Dzyubenko E Borbor M Vasileiadou P Kleinschnitz C Roll L. Tenascin-C Preserves Microglia Surveillance and Restricts Leukocyte and, More Specifically, T Cell Infiltration of the Ischemic Brain. Brain Behav Immun (2020) 91:639–48. 10.1016/j.bbi.2020.10.016 33122023

[50] Ummarino D. Systemic Sclerosis: Tenascin C Perpetuates Tissue Fibrosis. Nat Rev Rheumatol (2016) 12:375. 10.1038/nrrheum.2016.99 27278125

[51] Sun Z Velazquez-Quesada I Murdamoothoo D Ahowesso C Yilmaz A Spenle C. Tenascin-C Increases Lung Metastasis by Impacting Blood Vessel Invasions. Matrix Biol (2019) 83:26–47. 10.1016/j.matbio.2019.07.001 31288084

[52] Kasprzycka M Hammarstrom C Haraldsen G. Tenascins in Fibrotic Disorders-From Bench to Bedside. Cell Adh Migr (2015) 9:83–9. 10.4161/19336918.2014.994901 PMC4594616 25793575

[53] Katoh D Kozuka Y Noro A Ogawa T Imanaka-Yoshida K Yoshida T. Tenascin-C Induces Phenotypic Changes in Fibroblasts to Myofibroblasts With High Contractility Through the Integrin αvβ1/Transforming Growth Factor β/SMAD Signaling Axis in Human Breast Cancer. Am J Pathol (2020) 190(10):2123–35. 10.1016/j.ajpath.2020.06.008 32650003

[54] Midwood K Sacre S Piccinini AM Inglis J Trebaul A Chan E. Tenascin-C is an Endogenous Activator of Toll-Like Receptor 4 That is Essential for Maintaining Inflammation in Arthritic Joint Disease. Nat Med (2009) 15:774–80. 10.1038/nm.1987 19561617

[55] Yeo SY Lee KW Shin D An S Cho KH Kim SH. A Positive Feedback Loop Bi-Stably Activates Fibroblasts. Nat Commun (2018) 9:3016. 10.1038/s41467-018-05274-6 30069061 PMC6070563

[56] Langlois B Saupe F Rupp T Arnold C van der Heyden M Orend G. Angiomatrix, a Signature of the Tumor Angiogenic Switch-Specific Matrisome, Correlates With Poor Prognosis for Glioma and Colorectal Cancer Patients. Oncotarget (2014) 5:10529–45. 10.18632/oncotarget.2470 PMC4279391 25301723

[57] Mhaidly R Mechta-Grigoriou F. Fibroblast Heterogeneity in Tumor Micro-Environment: Role in Immunosuppression and New Therapies. Semin Immunol (2020) 48:101417. 10.1016/j.smim.2020.101417 33077325

[58] Schmid P Cortes J Pusztai L McArthur H Kümmel S Bergh J. Pembrolizumab for Early Triple-Negative Breast Cancer. N Engl J Med (2020) 382:810–21. 10.1056/NEJMoa1910549 32101663

[59] Yalcin F Dzaye O Xia S. Tenascin-C Function in Glioma: Immunomodulation and Beyond. Adv Exp Med Biol (2020) 1272:149–172. 10.1007/978-3-030-48457-6_9 32845507

[60] Li ZL Cortes J Pusztai L McArthur H Kümmel S Bergh J. Autophagy Deficiency Promotes Triple-Negative Breast Cancer Resistance to T Cell-Mediated Cytotoxicity by Blocking Tenascin-C Degradation. Nat Commun (2020) 11(1):3806. 10.1038/s41467-020-17395-y 32732922 PMC7393512

[61] Hauzenberger D Bergström SE Klominek J Sundqvist KG. Spectrum of Extracellular Matrix Degrading Enzymes in Normal and Malignant T Lymphocytes. Anticancer Res (1999) 19(3A):1945–52. 10470139

[62] Benbow JH Thompson KJ Cope HL Brandon-Warner E Culberson CR Bossi KL. Diet-Induced Obesity Enhances Progression of Hepatocellular Carcinoma Through Tenascin-C/Toll-Like Receptor 4 Signaling. Am J Pathol (2016) 186(1):145–58. 10.1016/j.ajpath.2015.09.015 26603137

[63] Deligne C Murdamoothoo D Gammage AN Gschwandtner M Erne W Loustau T. Matrix-Targeting Immunotherapy Controls Tumor Growth and Spread by Switching Macrophage Phenotype. Cancer Immunol Res (2020) 8:368–82. 10.1158/2326-6066.CIR-19-0276 PMC7611136 31941671

[64] Spenlé C Loustau T Murdamoothoo D Erne W Beghelli-de la Forest Divonne S Veber R. Tenascin-C Orchestrates an Immune-Suppressive Tumor Microenvironment in Oral Squamous Cell Carcinoma. Cancer Immunol Res (2020) 8:1122–38. 10.1158/2326-6066.CIR-20-0074 32665262

[65] Golledge J Clancy P Maguire J Lincz L Koblar S. The Role of Tenascin C in Cardiovascular Disease. Cardiovasc Res (2011) 92:19–28. 10.1093/cvr/cvr183 21712412

[66] Imanaka-Yoshida K Aoki H. Tenascin-C and Mechanotransduction in the Development and Diseases of Cardiovascular System. Front Physiol (2014) 5:283. 10.3389/fphys.2014.00283 25120494 PMC4114189

[67] Imanaka-Yoshida K Tawara I Yoshida T. Tenascin-C in Cardiac Disease: A Sophisticated Controller of Inflammation, Repair, and Fibrosis. Am J Physiol Physiol (2020) 319(5):C781–C796. 10.1152/ajpcell.00353.2020 32845719

[68] Gao W Li J Ni H Shi H Qi Z Zhu S. Tenascin C: A Potential Biomarker for Predicting the Severity of Coronary Atherosclerosis. J Atheroscler Thromb (2019) 26(1):31–8. 10.5551/jat.42887 PMC6308263 29769455

[69] Liu R He Y Li B Liu J Ren Y Han W. Tenascin-C Produced by Oxidized LDL-Stimulated Macrophages Increases Foam Cell Formation Through Toll-Like Receptor-4. Mol Cells (2012) 34(1):35–41. 10.1007/s10059-012-0054-x 22699754 PMC3887780

[70] Steffensen LB Mortensen MB Kjolby M Hagensen MK Oxvig C Bentzon JF. Disturbed Laminar Blood Flow Vastly Augments Lipoprotein Retention in the Artery Wall: A Key Mechanism Distinguishing Susceptible From Resistant Sites. Arterioscler Thromb Vasc Biol (2015) 35:1928–35. 10.1161/ATVBAHA.115.305874 26183617

[71] Wang L Wang W Shah PK Song L Yang M Sharifi BG. Deletion of Tenascin-C Gene Exacerbates Atherosclerosis and Induces Intraplaque Hemorrhage in Apo-E-Deficient Mice. Cardiovasc Pathol (2012) 21(5):398–413. 10.1016/j.carpath.2011.12.005 22300502 PMC3345312

[72] Distler JHW Gyorfi AH Ramanujam M Whitfield ML Konigshoff M Lafyatis R. Shared and Distinct Mechanisms of Fibrosis. Nat Rev Rheumatol (2019) 15:705–30. 10.1038/s41584-019-0322-7 31712723

[73] Mack M. Inflammation and Fibrosis. Matrix Biol (2018) 68–69:106–21. 10.1016/j.matbio.2017.11.010 29196207

[74] Suzuki H Fujimoto M Kawakita F Liu L Nakano F Nishikawa H. Toll-Like Receptor 4 and Tenascin-C Signaling in Cerebral Vasospasm and Brain Injuries After Subarachnoid Hemorrhage. Acta Neurochir Suppl (2020) 127:91–6. 10.1007/978-3-030-04615-6_15 31407069

[75] El-Karef A Yoshida T Gabazza EC Nishioka T Inada H Sakakura T. Deficiency of Tenascin-C Attenuates Liver Fibrosis in Immune-Mediated Chronic Hepatitis in Mice. J Pathol (2007) 211(1):86–94. 10.1002/path.2099 17121418

[76] Zhu H Liao J Zhou X Hong X Song D Hou FF. Tenascin-C Promotes Acute Kidney Injury to Chronic Kidney Disease Progression by Impairing Tubular Integrity Via $α$V$β$6 Integrin Signaling. Kidney Int (2020) 97(5):1017–31. 10.1016/j.kint.2020.01.026 PMC8112450 32245660

[77] Ricard-Blum S. The Collagen Family. Cold Spring Harb Perspect Biol (2011). 10.1101/cshperspect.a004978 PMC3003457 21421911

[78] Kadler KE Baldock C Bella J Boot-Handford RP. Collagens At a Glance. J Cell Sci (2007) 120(Pt 12):1955–8. 10.1242/jcs.03453 17550969

[79] Gordon MK Hahn RA. Collagens. Cell Tissue Res (2010) 339:247–57. 10.1007/s00441-009-0844-4 PMC2997103 19693541

[80] Gelse K Pöschl E Aigner T. Collagens - Structure, Function, and Biosynthesis. Adv Drug Deliv Rev (2003) 55(12):1531–46. 10.1016/j.addr.2003.08.002 14623400

[81] Gomez-Navarro N Melero A Li XH Boulanger J Kukulski W Miller EA. Cargo Crowding Contributes to Sorting Stringency in COPII Vesicles. J Cell Biol (2020) 219(7):e201806038. 10.1083/JCB.201806038 32406500 PMC7300426

[82] Gomez-Navarro N Miller EA. COP-Coated Vesicles. Curr Biol (2016). 10.1016/j.cub.2015.12.017 26811885

[83] McCaughey J Stephens DJ. ER-to-Golgi Transport: A Sizeable Problem. Trends Cell Biol (2019) 29:940–53. 10.1016/j.tcb.2019.08.007 31630879

[84] Malhotra V Erlmann P. The Pathway of Collagen Secretion. Annu Rev Cell Dev Biol (2015) 31:109–24. 10.1146/annurev-cellbio-100913-013002 26422332

[85] Raote I Ortega Bellido M Pirozzi M Zhang C Melville D Parashuraman S. TANGO1 assembles into rings around COPII coats at ER exit sitess. J Cell Biol (2017) 216:901–9. 10.1083/jcb.201608080 PMC5379947 28280121

[86] Tanabe T Maeda M Saito K Katada T. Dual Function of Ctage5 in Collagen Export From the Endoplasmic Reticulum. Mol Biol Cell (2016) 27(13):2008–13. 10.1091/mbc.E16-03-0180 PMC4927275 27170179

[87] Jin L Pahuja KB Wickliffe KE Gorur A Baumgärtel C Schekman R. Ubiquitin-Dependent Regulation of COPII Coat Size and Function. Nature (2012) 482(7386):495–500. 10.1038/nature10822 22358839 PMC3292188

[88] Al-Yafeai Z Yurdagul A Peretik JM Alfaidi M Murphy PA Orr AW. Endothelial FN (Fibronectin) Deposition by α5β1 Integrins Drives Atherogenic Inflammation. Arterioscler Thromb Vasc Biol (2018) 38:2601–14. 10.1161/ATVBAHA.118.311705 PMC6209122 30354234

[89] Kii I Nishiyama T Kudo A. Periostin Promotes Secretion of Fibronectin From the Endoplasmic Reticulum. Biochem Biophys Res Commun (2016) 470:888–93. 10.1016/j.bbrc.2016.01.139 26820539

[90] Choi MG Hynes RO. Biosynthesis and Processing of Fibronectin in NIL.8 Hamster Cells. J Biol Chem (1979) 254:12050–5. 10.1016/S0021-9258(19)86426-8 500694

[91] Mosher DF Fogerty FJ Chernousov MA Barry EL. Assembly of Fibronectin Into Extracellular Matrix. Ann N Y Acad Sci (1991) 614:167–80. 10.1111/j.1749-6632.1991.tb43701.x 1673833

[92] Uchida N Smilowitz H Ledger PW Tanzer ML. Kinetic Studies of the Intracellular Transport of Procollagen and Fibronectin in Human Fibroblasts. Effects of the Monovalent Ionophore, Monensin. J Biol Chem (1980) 255:8638–44. 10.1016/S0021-9258(18)43547-8 7410384

[93] Pizzey JA Bennett FA Jones GE. Monensin Inhibits Initial Spreading of Cultured Human Fibroblasts. Nature (1983) 305:315–7. 10.1038/305315a0 6621684

[94] Villiger B Kelley DG Engleman W Kuhn C 3rd Mcdonald JA. Human Alveolar Macrophage Fibronectin: Synthesis, Secretion, and Ultrastructural Localization During Gelatin-Coated Latex Particle Binding. J Cell Biol (1981) 90:711–20. 10.1083/jcb.90.3.711 PMC2111908 7287821

[95] Hedman K. Intracellular Localization of Fibronectin Using Immunoperoxidase Cytochemistry in Light and Electron Microscopy. J Histochem Cytochem (1980) 28:1233–41. 10.1177/28.11.7000891 7000891

[96] Ledger PW Uchida N Tanzer ML. Immunocytochemical Localization of Procollagen and Fibronectin in Human Fibroblasts: Effects of the Monovalent Ionophore, Monensin. J Cell Biol (1980) 87:663–71. 10.1083/jcb.87.3.663 PMC2110792 7007394

[97] Yamada SS Yamada KM Willingham MC. Intracellular Localization of Fibronectin by Immunoelectron Microscopy. J Histochem Cytochem (1980) 28:953–60. 10.1177/28.9.6997370 6997370

[98] Anderson RG Pathak RK. Vesicles and Cisternae in the Trans Golgi Apparatus of Human Fibroblasts are Acidic Compartments. Cell (1985) 40:635–43. 10.1016/0092-8674(85)90212-0 3882239

[99] Zhu M Tao J Vasievich MP Wei W Zhu G Khoriaty RN. Neural Tube Opening and Abnormal Extraembryonic Membrane Development in SEC23A Deficient Mice. Sci Rep (2015) 5:15471. 10.1038/srep15471 26494538 PMC4616029

[100] Sarmah S Barrallo-Gimeno A Melville DB Topczewski J Solnica-Krezel L Knapik EW. Sec24D-Dependent Transport of Extracellular Matrix Proteins is Required for Zebrafish Skeletal Morphogenesis. PLoS One (2010) 5:e10367. 10.1371/journal.pone.0010367 20442775 PMC2860987

[101] Wilson DG Phamluong K Li L Sun M Cao TC Liu PS. Global Defects in Collagen Secretion in a Mia3/TANGO1 Knockout Mouse. J Cell Biol (2011) 193:935–51. 10.1083/jcb.201007162 PMC3105544 21606205

[102] Hou W Jerome-Majewska LA. TMED2/Emp24 is Required in Both the Chorion and the Allantois for Placental Labyrinth Layer Development. Dev Biol (2018) 444:20–32. 10.1016/j.ydbio.2018.09.012 30236446

[103] Redick SD Schwarzbauer JE. Rapid Intracellular Assembly of Tenascin Hexabrachions Suggests a Novel Cotranslational Process. J Cell Sci (1995) 108:1761–9. 10.1242/jcs.108.4.1761 7542260

[104] Kammerer RA Schulthess T Landwehr R Lustig A Fischer D Engel J. Tenascin-C Hexabrachion Assembly is a Sequential Two-Step Process Initiated by Coiled-Coil Alpha-Helices. J Biol Chem (1998) 273:10602–8. 10.1074/jbc.273.17.10602 9553121

[105] Caubit X Riou JF Coulon J Arsanto JP Benraiss A Boucaut JC. Tenascin Expression in Developing, Adult and Regenerating Caudal Spinal Cord in the Urodele Amphibians. Int J Dev Biol (1994) 38:661–72 10.3402/jev.v4.27066. 7540033

[106] Teng J Zhang PL Russell WJ Zheng LP Jones ML Herrera GA. Insights Into Mechanisms Responsible for Mesangial Alterations Associated With Fibrogenic Glomerulopathic Light Chains. Nephron Physiol (2003) 94:28–38. 10.1159/000071288 12845220

[107] Becker A Thakur BK Weiss JM Kim HS Peinado H Lyden D. Extracellular Vesicles in Cancer: Cell-to-Cell Mediators of Metastasis. Cancer Cell (2016) 30(6):836–48. 10.1016/j.ccell.2016.10.009 PMC5157696 27960084

[108] Albacete-Albacete L Navarro-Lérida I López JA Martín-Padura I Astudillo AM Ferrarini A. ECM Deposition is Driven by Caveolin-1–Dependent Regulation of Exosomal Biogenesis and Cargo Sorting. J Cell Biol (2020) 219(11):e202006178. 10.1083/jcb.202006178 33053168 PMC7551399

[109] Sung BH Ketova T Hoshino D Zijlstra A Weaver AM. Directional Cell Movement Through Tissues is Controlled by Exosome Secretion. Nat Commun (2015) 6:7164. 10.1038/ncomms8164 25968605 PMC4435734

[110] van Niel G D’Angelo G Raposo G. Shedding Light on the Cell Biology of Extracellular Vesicles. Nat Rev Mol Cell Biol (2018) 19:213–28. 10.1038/nrm.2017.125 29339798

[111] Colombo M Raposo G Théry C. Biogenesis, Secretion, and Intercellular Interactions of Exosomes and Other Extracellular Vesicles. Annu Rev Cell Dev Biol (2014) 30:255–89. 10.1146/annurev-cellbio-101512-122326 25288114

[112] Mathieu M Martin-Jaular L Lavieu G Théry C. Specificities of Secretion and Uptake of Exosomes and Other Extracellular Vesicles for Cell-to-Cell Communication. Nat Cell Biol (2019) 21:9–17. 10.1038/s41556-018-0250-9 30602770

[113] Villarroya-Beltri C Baixauli F Gutiérrez-Vázquez C Sánchez-Madrid F Mittelbrunn M. Sorting it Out: Regulation of Exosome Loading. Semin Cancer Biol (2014) 28:3–13. 10.1016/j.semcancer.2014.04.009 24769058 PMC4640178

[114] Henne WM Buchkovich NJ Emr SD. The Escrt Pathway. Dev Cell (2011) 21(1):77–91. 10.1016/j.devcel.2011.05.015 21763610

[115] Williams RL Urbé S. The Emerging Shape of the ESCRT Machinery. Nat Rev Mol Cell Biol (2007) 8:355–68. 10.1038/nrm2162 17450176

[116] Raiborg C Stenmark H. The ESCRT Machinery in Endosomal Sorting of Ubiquitylated Membrane Proteins. Nature (2009) 458:445–52. 10.1038/nature07961 19325624

[117] Vietri M Radulovic M Stenmark H. The Many Functions of Escrts. Nat Rev Mol Cell Biol (2020) 21:25–42. 10.1038/s41580-019-0177-4 31705132

[118] Stuffers S Sem Wegner C Stenmark H Brech A. Multivesicular Endosome Biogenesis in the Absence of Escrts. Traffic (2009) 10:925–37. 10.1111/j.1600-0854.2009.00920.x 19490536

[119] Trajkovic K Hsu C Chiantia S Rajendran L Wenzel D Wieland F. Ceramide Triggers Budding of Exosome Vesicles Into Multivesicular Endosomes. Science (80-) (2008). 10.1126/science.1153124 18309083

[120] Gruenberg J. Life in the Lumen: The Multivesicular Endosome. Traffic (2020) 21(1):76–93. 10.1111/tra.12715 31854087 PMC7004041

[121] Andreu Z Yáñez-Mó M. Tetraspanins in Extracellular Vesicle Formation and Function. Front Immunol (2014) 5(442):442. 10.3389/fimmu.2014.00442 25278937 PMC4165315

[122] Campos A Salomon C Bustos R Diaz J Martinez S Silva V. Caveolin-1-Containing Extracellular Vesicles Transport Adhesion Proteins and Promote Malignancy in Breast Cancer Cell Lines. Nanomedicine (Lond) (2018) 13:2597–609. 10.2217/nnm-2018-0094 30338706

[123] Ni K Wang C Carnino JM Jin Y. The Evolving Role of Caveolin-1: A Critical Regulator of Extracellular Vesicles. Med Sci (Basel Switzerland) (2020) 8(4):46. 10.3390/medsci8040046 PMC7712126 33158117

[124] Li B Antonyak MA Zhang J Cerione RA. Rhoa Triggers a Specific Signaling Pathway That Generates Transforming Microvesicles in Cancer Cells. Oncogene (2012) 31(45):4740–9. 10.1038/onc.2011.636 PMC3607381 22266864

[125] Yanez-Mo M Siljander PR Andreu Z Zavec AB Borras FE Buzas EI. Biological Properties of Extracellular Vesicles and Their Physiological Functions. J Extracell Vesicles (2015) 4:27066. 25979354 10.3402/jev.v4.27066 PMC4433489

[126] Raposo G Nijman HW Stoorvogel W Liejendekker R Harding CV Melief CJ. B Lymphocytes Secrete Antigen-Presenting Vesicles. J Exp Med (1996) 183:1161–72. 10.1084/jem.183.3.1161 PMC2192324 8642258

[127] Greening DW Gopal SK Xu R Simpson RJ Chen W. Exosomes and Their Roles in Immune Regulation and Cancer. Semin Cell Dev Biol (2015) 40:72–81. 10.1016/j.semcdb.2015.02.009 25724562

[128] Thery C Amigorena S. The Cell Biology of Antigen Presentation in Dendritic Cells. Curr Opin Immunol (2001) 13:45–51. 10.1016/S0952-7915(00)00180-1 11154916

[129] Mittelbrunn M Gutiérrez-Vázquez C Villarroya-Beltri C González S Sánchez-Cabo F González MÁ. Unidirectional Transfer of Microrna-Loaded Exosomes From T Cells to Antigen-Presenting Cells. Nat Commun (2011) 2(282). 10.1038/ncomms1285 PMC3104548 21505438

[130] Fernandez-Messina L Rodriguez-Galan A de Yebenes VG Gutierrez-Vazquez C Tenreiro S Seabra MC. Transfer of Extracellular Vesicle-Microrna Controls Germinal Center Reaction and Antibody Production. EMBO Rep (2020) 21:e48925. 10.15252/embr.201948925 32073750 PMC7132182

[131] Fernandez-Messina L Gutierrez-Vazquez C Rivas-Garcia E Sanchez-Madrid F de la Fuente H. Immunomodulatory Role of Micrornas Transferred by Extracellular Vesicles. Biol Cell (2015) 107:61–77. 10.1111/boc.201400081 25564937 PMC5010100

[132] MacKenzie A Wilson HL Kiss-Toth E Dower SK North RA Surprenant A. Rapid Secretion of Interleukin-1beta by Microvesicle Shedding. Immunity (2001) 15:825–35. 10.1016/S1074-7613(01)00229-1 11728343

[133] Pizzirani C Ferrari D Chiozzi P Adinolfi E Sandona D Savaglio E. Stimulation of P2 Receptors Causes Release of IL-1beta-Loaded Microvesicles From Human Dendritic Cells. Blood (2007) 109:3856–64. 10.1182/blood-2005-06-031377 17192399

[134] Dower SK Kronheim SR Hopp TP Cantrell M Deeley M Gillis S. The Cell Surface Receptors for Interleukin-1 Alpha and Interleukin-1 Beta are Identical. Nature (1986) 324:266–8. 10.1038/324266a0 2946959

[135] Borthwick LA. The IL-1 Cytokine Family and its Role in Inflammation and Fibrosis in the Lung. Semin Immunopathol (2016) 38:517–34. 10.1007/s00281-016-0559-z PMC4896974 27001429

[136] Kandere-Grzybowska K Letourneau R Kempuraj D Donelan J Poplawski S Boucher W. IL-1 Induces Vesicular Secretion of IL-6 Without Degranulation From Human Mast Cells. J Immunol (2003) 171:4830–6. 10.4049/jimmunol.171.9.4830 14568962

[137] Femmino S Penna C Margarita S Comita S Brizzi MF Pagliaro P. Extracellular Vesicles and Cardiovascular System: Biomarkers and Cardioprotective Effectors. Vasc Pharmacol (2020) 135:106790. 10.1016/j.vph.2020.106790 32861822

[138] Wang R Ding Q Yaqoob U de Assuncao TM Verma VK Hirsova P. Exosome Adherence and Internalization by Hepatic Stellate Cells Triggers Sphingosine 1-Phosphate-Dependent Migration. J Biol Chem (2015) 290:30684–96. 10.1074/jbc.M115.671735 PMC4692200 26534962

[139] Lawson C Vicencio JM Yellon DM Davidson SM. Microvesicles and Exosomes: New Players in Metabolic and Cardiovascular Disease. J Endocrinol (2016) 228:R57–71. 10.1530/JOE-15-0201 26743452

[140] Vanhaverbeke M Gal D Holvoet P. Functional Role of Cardiovascular Exosomes in Myocardial Injury and Atherosclerosis. Adv Exp Med Biol (2017) 998:45–58. 10.1007/978-981-10-4397-0_3 28936731

[141] Hergenreider E Heydt S Treguer K Boettger T Horrevoets AJ Zeiher AM. Atheroprotective Communication Between Endothelial Cells and Smooth Muscle Cells Through Mirnas. Nat Cell Biol (2012) 14:249–56. 10.1038/ncb2441 22327366

[142] Li B Zang G Zhong W Chen R Zhang Y Yang P. Activation of CD137 Signaling Promotes Neointimal Formation by Attenuating TET2 and Transferrring From Endothelial Cell-Derived Exosomes to Vascular Smooth Muscle Cells. BioMed Pharmacother (2020) 121:109593. 10.1016/j.biopha.2019.109593 31766102

[143] Zheng B Yin WN Suzuki T Zhang XH Zhang Y Song LL. Exosome-Mediated Mir-155 Transfer From Smooth Muscle Cells to Endothelial Cells Induces Endothelial Injury and Promotes Atherosclerosis. Mol Ther (2017) 25:1279–94. 10.1016/j.ymthe.2017.03.031 PMC5475247 28408180

[144] Huang C Han J Wu Y Li S Wang Q Lin W. Exosomal MALAT1 Derived From Oxidized Low-Density Lipoprotein-Treated Endothelial Cells Promotes M2 Macrophage Polarization. Mol Med Rep (2018) 18:509–15. 10.3892/mmr.2018.8982 29750307

[145] Gao H Wang X Lin C An Z Yu J Cao H. Exosomal MALAT1 Derived From Ox-LDL-Treated Endothelial Cells Induce Neutrophil Extracellular Traps to Aggravate Atherosclerosis. Biol Chem (2020) 401:367–76. 10.1515/hsz-2019-0219 31318684

[146] Tang N Sun B Gupta A Rempel H Pulliam L. Monocyte Exosomes Induce Adhesion Molecules and Cytokines Via Activation of NF-Kappab in Endothelial Cells. FASEB J (2016) 30:3097–106. 10.1096/fj.201600368RR PMC5001509 27226520

[147] Kapustin AN Chatrou ML Drozdov I Zheng Y Davidson SM Soong D. Vascular Smooth Muscle Cell Calcification is Mediated by Regulated Exosome Secretion. Circ Res (2015) 116:1312–23. 10.1161/CIRCRESAHA.116.305012 25711438

[148] Cheng Y Wang X Yang J Duan X Yao Y Shi X. A Translational Study of Urine Mirnas in Acute Myocardial Infarction. J Mol Cell Cardiol (2012) 53:668–76. 10.1016/j.yjmcc.2012.08.010 PMC4492106 22921780

[149] Aurora AB Mahmoud AI Luo X Johnson BA van Rooij E Matsuzaki S. Microrna-214 Protects the Mouse Heart From Ischemic Injury by Controlling Ca(2)(+) Overload and Cell Death. J Clin Invest (2012) 122:1222–32. 10.1172/JCI59327 PMC3314458 22426211

[150] Prasetyanti PR Medema JP. Intra-Tumor Heterogeneity From a Cancer Stem Cell Perspective. Mol Cancer (2017) 16:41. 10.1186/s12943-017-0600-4 28209166 PMC5314464

[151] McGranahan N Swanton C. Clonal Heterogeneity and Tumor Evolution: Past, Present, and the Future. Cell (2017) 168:613–28. 10.1016/j.cell.2017.01.018 28187284

[152] Meacham CE Morrison SJ. Tumour Heterogeneity and Cancer Cell Plasticity. Nature (2013) 501:328–37. 10.1038/nature12624 PMC4521623 24048065

[153] Demory Beckler M Higginbotham JN Franklin JL Ham AJ Halvey PJ Imasuen IE. Proteomic Analysis of Exosomes From Mutant KRAS Colon Cancer Cells Identifies Intercellular Transfer of Mutant KRAS. Mol Cell Proteomics (2013) 12:343–55. 10.1074/mcp.M112.022806 PMC3567858 23161513

[154] Skog J Wurdinger T van Rijn S Meijer DH Gainche L Sena-Esteves M. Glioblastoma Microvesicles Transport RNA and Proteins That Promote Tumour Growth and Provide Diagnostic Biomarkers. Nat Cell Biol (2008) 10:1470–6. 10.1038/ncb1800 PMC3423894 19011622

[155] Khan S Jutzy JM Aspe JR McGregor DW Neidigh JW Wall NR. Survivin is Released From Cancer Cells Via Exosomes. Apoptosis (2011) 16:1–12. 10.1007/s10495-010-0534-4 20717727 PMC3174681

[156] Kalluri R. Basement Membranes: Structure, Assembly and Role in Tumour Angiogenesis. Nat Rev Cancer (2003) 3:422–33. 10.1038/nrc1094 12778132

[157] De Palma M Biziato D Petrova TV. Microenvironmental Regulation of Tumour Angiogenesis. Nat Rev Cancer (2017) 17:457–74. 10.1038/nrc.2017.51 28706266

[158] Mashouri L Yousefi H Aref AR Ahadi AM Molaei F Alahari SK. Exosomes: Composition, Biogenesis, and Mechanisms in Cancer Metastasis and Drug Resistance. Mol Cancer (2019) 18:75. 10.1186/s12943-019-0991-5 30940145 PMC6444571

[159] Zhou W Fong MY Min Y Somlo G Liu L Palomares MR. Cancer-Secreted Mir-105 Destroys Vascular Endothelial Barriers to Promote Metastasis. Cancer Cell (2014) 25:501–15. 10.1016/j.ccr.2014.03.007 PMC4016197 24735924

[160] Peinado H Aleckovic M Lavotshkin S Matei I Costa-Silva B Moreno-Bueno G. Melanoma Exosomes Educate Bone Marrow Progenitor Cells Toward a Pro-Metastatic Phenotype Through MET. Nat Med (2012) 18:883–91. 10.1038/nm.2753 PMC3645291 22635005

[161] Zeng Z Li Y Pan Y Lan X Song F Sun J. Cancer-Derived Exosomal Mir-25-3p Promotes Pre-Metastatic Niche Formation by Inducing Vascular Permeability and Angiogenesis. Nat Commun (2018) 9:5395. 10.1038/s41467-018-07810-w 30568162 PMC6300604

[162] Viaud S Terme M Flament C Taieb J Andre F Novault S. Dendritic Cell-Derived Exosomes Promote Natural Killer Cell Activation and Proliferation: A Role for NKG2D Ligands and IL-15Ralpha. PLoS One (2009) 4:e4942. 10.1371/journal.pone.0004942 19319200 PMC2657211

[163] Obregon C Rothen-Rutishauser B Gerber P Gehr P Nicod LP. Active Uptake of Dendritic Cell-Derived Exovesicles by Epithelial Cells Induces the Release of Inflammatory Mediators Through a TNF-Alpha-Mediated Pathway. Am J Pathol (2009) 175:696–705. 10.2353/ajpath.2009.080716 19628765 PMC2715287

[164] Gastpar R Gehrmann M Bausero MA Asea A Gross C Schroeder JA. Heat Shock Protein 70 Surface-Positive Tumor Exosomes Stimulate Migratory and Cytolytic Activity of Natural Killer Cells. Cancer Res (2005) 65:5238–47. 10.1158/0008-5472.CAN-04-3804 PMC1785299 15958569

[165] Kalluri R. The Biology and Function of Fibroblasts in Cancer. Nat Rev Cancer (2016) 16:582–98. 10.1038/nrc.2016.73 27550820

[166] Wortzel I Dror S Kenific CM Lyden D. Exosome-Mediated Metastasis: Communication From a Distance. Dev Cell (2019) 49:347–60. 10.1016/j.devcel.2019.04.011 31063754

[167] Yang F Ning Z Ma L Liu W Shao C Shu Y. Exosomal Mirnas and Mirna Dysregulation in Cancer-Associated Fibroblasts. Mol Cancer (2017) 16:148. 10.1186/s12943-017-0718-4 28851377 PMC5576273

[168] Hoshino A Costa-Silva B Shen TL Rodrigues G Hashimoto A Tesic Mark M. Tumour Exosome Integrins Determine Organotropic Metastasis. Nature (2015) 527:329–35 10.1038/nature15756. PMC4788391 26524530

[169] Costa-Silva B Aiello NM Ocean AJ Singh S Zhang H Thakur BK. Pancreatic Cancer Exosomes Initiate Pre-Metastatic Niche Formation in the Liver. Nat Cell Biol (2015) 17:816–26 10.1038/ncb3169. PMC5769922 25985394

[170] Xu G Zhang B Ye J Cao S Shi J Zhao Y. Exosomal Mirna-139 in Cancer-Associated Fibroblasts Inhibits Gastric Cancer Progression by Repressing MMP11 Expression. Int J Biol Sci (2019) 15:2320–9. 10.7150/ijbs.33750 PMC6775321 31595150

[171] Chen Z Wang H Xia Y Yan F Lu Y. Therapeutic Potential of Mesenchymal Cell-Derived Mirna-150-5p-Expressing Exosomes in Rheumatoid Arthritis Mediated by the Modulation of MMP14 and VEGF. J Immunol (2018) 201:2472–82. 10.4049/jimmunol.1800304 PMC6176104 30224512

[172] Rilla K Mustonen AM Arasu UT Harkonen K Matilainen J Nieminen P. Extracellular Vesicles are Integral and Functional Components of the Extracellular Matrix. Matrix Biol (2019) 75–76:201–19. 10.1016/j.matbio.2017.10.003 29066152

[173] Manou D. The Complex Interplay Between Extracellular Matrix and Cells in Tissues. Methods Mol Biol (2019) 1952:1–20. 10.1007/978-1-4939-9133-4_1 30825161

[174] Ramteke A Caon I Bouris P Triantaphyllidou IE Giaroni C Passi A. Exosomes Secreted Under Hypoxia Enhance Invasiveness and Stemness of Prostate Cancer Cells by Targeting Adherens Junction Molecules. Mol Carcinog (2015) 54:554–65. 10.1002/mc.22124 PMC4706761 24347249

[175] Li R Wang Y Zhang X Feng M Ma J Li J. Exosome-Mediated Secretion of LOXL4 Promotes Hepatocellular Carcinoma Cell Invasion and Metastasis. Mol Cancer (2019) 18:18. 10.1186/s12943-019-0948-8 30704479 PMC6354392

[176] de Jong OG van Balkom BW Gremmels H Verhaar MC. Exosomes From Hypoxic Endothelial Cells Have Increased Collagen Crosslinking Activity Through Up-Regulation of Lysyl Oxidase-Like 2. J Cell Mol Med (2016) 20:342–50. 10.1111/jcmm.12730 PMC4727569 26612622

[177] Shimoda M. Extracellular Vesicle-Associated Mmps: A Modulator of the Tissue Microenvironment. Adv Clin Chem (2019) 88:35–66. 10.1016/bs.acc.2018.10.006 30612606

[178] Nawaz M Shah N Zanetti B Maugeri M Silvestre R Fatima F. Extracellular Vesicles and Matrix Remodeling Enzymes: The Emerging Roles in Extracellular Matrix Remodeling, Progression of Diseases and Tissue Repair. Cells (2018) 7(10):167. 10.3390/cells7100167 PMC6210724 30322133

[179] Ali SY Sajdera SW Anderson HC. Isolation and Characterization of Calcifying Matrix Vesicles From Epiphyseal Cartilage. Proc Natl Acad Sci U S A (1970) 67:1513–20. 10.1073/pnas.67.3.1513 PMC283384 5274475

[180] Bonucci E. Fine Structure and Histochemistry of ‘Calcifying Globules’ in Epiphyseal Cartilage. Z Zellforsch Mikrosk Anat (1970) 103:192–217. 10.1007/BF00337312 5412827

[181] Shapiro IM Landis WJ Risbud MV. Matrix Vesicles: Are They Anchored Exosomes? Bone (2015) 79:29–36. 10.1016/j.bone.2015.05.013 25980744 PMC4501874

[182] Hasegawa T. Ultrastructure and Biological Function of Matrix Vesicles in Bone Mineralization. Histochem Cell Biol (2018) 149:289–304. 10.1007/s00418-018-1646-0 29411103

[183] Ji H Greening DW Barnes TW Lim JW Tauro BJ Rai A. Proteome Profiling of Exosomes Derived From Human Primary and Metastatic Colorectal Cancer Cells Reveal Differential Expression of Key Metastatic Factors and Signal Transduction Components. Proteomics (2013) 13:1672–86. 10.1002/pmic.201200562 23585443

[184] Zheng J Hernandez JM Doussot A Bojmar L Zambirinis CP Costa-Silva B. Extracellular Matrix Proteins and Carcinoembryonic Antigen-Related Cell Adhesion Molecules Characterize Pancreatic Duct Fluid Exosomes in Patients With Pancreatic Cancer. HPB (2018) 20:597–604. 10.1016/j.hpb.2017.12.010 29339034 PMC6779041

[185] Manickam G Moffatt P Murshed M. Role of SMPD3 During Bone Fracture Healing and Regulation of its Expression. Mol Cell Biol (2019) 39(4):e00370–18. 10.1128/MCB.00370-18 PMC6362318 30530524

[186] Stoffel W Hammels I Jenke B Schmidt-Soltau I Niehoff A. Neutral Sphingomyelinase 2 (SMPD3) Deficiency in Mice Causes Chondrodysplasia With Unimpaired Skeletal Mineralization. Am J Pathol (2019) 189:1831–45. 10.1016/j.ajpath.2019.05.008 31199918

[187] Purushothaman A Bandari SK Liu J Mobley JA Brown EE Sanderson RD. Fibronectin on the Surface of Myeloma Cell-Derived Exosomes Mediates Exosome-Cell Interactions. J Biol Chem (2016) 291:1652–63. 10.1074/jbc.M115.686295 PMC4722448 26601950

[188] Dismuke WM Klingeborn M Stamer WD. Mechanism of Fibronectin Binding to Human Trabecular Meshwork Exosomes and its Modulation by Dexamethasone. PLoS One (2016) 11:e0165326. 10.1371/journal.pone.0165326 27783649 PMC5081181

[189] Shi F Sottile J. Caveolin-1-Dependent Beta1 Integrin Endocytosis is a Critical Regulator of Fibronectin Turnover. J Cell Sci (2008) 121:2360–71. 10.1242/jcs.014977 PMC2587120 18577581

[190] Sung BH Zhu X Kaverina I Weaver AM. Cortactin Controls Cell Motility and Lamellipodial Dynamics by Regulating ECM Secretion. Curr Biol (2011) 21:1460–9. 10.1016/j.cub.2011.06.065 PMC3175319 21856159

[191] Benesh EC Miller PM Pfaltzgraff ER Grega-Larson NE Hager HA Sung BH. Bves and NDRG4 Regulate Directional Epicardial Cell Migration Through Autocrine Extracellular Matrix Deposition. Mol Biol Cell (2013) 24:3496–510. 10.1091/mbc.e12-07-0539 PMC3826988 24048452

[192] Sung BH Weaver AM. Exosome Secretion Promotes Chemotaxis of Cancer Cells. Cell Adh Migr (2017) 11:187–95. 10.1080/19336918.2016.1273307 PMC5351719 28129015

[193] Hoshino D Kirkbride KC Costello K Clark ES Sinha S Grega-Larson N. Exosome Secretion is Enhanced by Invadopodia and Drives Invasive Behavior. Cell Rep (2013) 5:1159–68. 10.1016/j.celrep.2013.10.050 PMC3873329 24290760

[194] Chanda D Otoupalova E Hough KP Locy ML Bernard K Deshane JS. Fibronectin on the Surface of Extracellular Vesicles Mediates Fibroblast Invasion. Am J Respir Cell Mol Biol (2019) 60:279–88. 10.1165/rcmb.2018-0062OC PMC6397976 30321056

[195] Yuan O Lin C Wagner J Archard JA Deng P Halmai J. Exosomes Derived From Human Primed Mesenchymal Stem Cells Induce Mitosis and Potentiate Growth Factor Secretion. Stem Cells Dev (2019) 28:398–409. 10.1089/scd.2018.0200 30638129 PMC6441283

[196] Antonyak MA Li B Boroughs LK Johnson JL Druso JE Bryant KL. Cancer Cell-Derived Microvesicles Induce Transformation by Transferring Tissue Transglutaminase and Fibronectin to Recipient Cells. Proc Natl Acad Sci U S A (2011) 108:4852–7. 10.1073/pnas.1017667108 PMC3064359 21368175

[197] Osawa S Kurachi M Yamamoto H Yoshimoto Y Ishizaki Y. Fibronectin on Extracellular Vesicles From Microvascular Endothelial Cells is Involved in the Vesicle Uptake Into Oligodendrocyte Precursor Cells. Biochem Biophys Res Commun (2017) 488:232–8. 10.1016/j.bbrc.2017.05.049 28499870

[198] Bin BH Kim DK Kim NH Choi EJ Bhin J Kim ST. Fibronectin-Containing Extracellular Vesicles Protect Melanocytes Against Ultraviolet Radiation-Induced Cytotoxicity. J Invest Dermatol (2016) 136:957–66. 10.1016/j.jid.2015.08.001 26854492

[199] Deng Z Cheng Z Xiang X Yan J Zhuang X Liu C. Tumor Cell Cross Talk With Tumor-Associated Leukocytes Leads to Induction of Tumor Exosomal Fibronectin and Promotes Tumor Progression. Am J Pathol (2012) 180:390–8. 10.1016/j.ajpath.2011.09.023 22067905

[200] Atay S Gercel-Taylor C Taylor DD. Human Trophoblast-Derived Exosomal Fibronectin Induces Pro-Inflammatory IL-1beta Production by Macrophages. Am J Reprod Immunol (2011) 66:259–69. 10.1111/j.1600-0897.2011.00995.x 21410811

[201] Kulkarni R Prasad A. Exosomes Derived From HIV-1 Infected Dcs Mediate Viral Trans-Infection Via Fibronectin and Galectin-3. Sci Rep (2017) 7(1):14787. 10.1038/s41598-017-14817-8 29093555 PMC5665889

[202] Sharma M Kaur H Roy S. Tissue-Associated Self-Antigens Containing Exosomes: Role in Allograft Rejection. Hum Immunol (2018) 79:653–8. 10.1016/j.humimm.2018.06.005 PMC6098724 29908844

[203] Parton RG Del Pozo MA. Caveolae as Plasma Membrane Sensors, Protectors and Organizers. Nat Rev Mol Cell Biol (2013) 14(2):98–112. 10.1038/nrm3512 23340574

[204] Moreno-Vicente R Pavón DM Martín-Padura I Català-Montoro M Díez-Sánchez A Quílez-Álvarez A. Caveolin-1 Modulates Mechanotransduction Responses to Substrate Stiffness Through Actin-Dependent Control of YAP. Cell Rep (2018) 25(6):1622–1635.e6. 10.1016/j.celrep.2018.10.024 30404014 PMC6231326

[205] Pol A Morales-Paytuví F Bosch M Parton RG. Non-Caveolar Caveolins - Duties Outside the Caves. J Cell Sci (2020) 133(9):jcs241562. 10.1242/jcs.241562 32393675

[206] Enrich C Rentero C Grewal T Futter CE Eden ER. Cholesterol Overload: Contact Sites to the Rescue! Contact (2019). 10.1177/2515256419893507 PMC6923141 31858076

[207] Höglinger D Burgoyne T Sanchez-Heras E Hartwig P Colaco A Newton J. NPC1 Regulates ER Contacts With Endocytic Organelles to Mediate Cholesterol Egress. Nat Commun (2019) 10(1):4276. 10.1038/s41467-019-12152-2 31537798 PMC6753064

[208] Strippoli R Burgoyne T Sanchez-Heras E Hartwig P Colaco A Newton J. Caveolin1 and YAP Drive Mechanically Induced Mesothelial to Mesenchymal Transition and Fibrosis. Cell Death Dis (2020) 11(8):647. 10.1038/s41419-020-02822-1 32811813 PMC7435273

[209] Bissell MJ Hall HG Parry G. How Does the Extracellular Matrix Direct Gene Expression? *J* . Theor Biol (1982) 99:31–68. 10.1016/0022-5193(82)90388-5 6892044

[210] Strippoli R Sandoval P Moreno-Vicente R Rossi L Battistelli C Terri M. Molecular Mechanisms Underlying Peritoneal EMT and Fibrosis. Stem Cells Int (2016) 2016:3543678. 10.1155/2016/3543678 26941801 PMC4752998

[211] Inder KL Ruelcke JE Petelin L Moon H Choi E Rae J. Cavin-1/PTRF Alters Prostate Cancer Cell-Derived Extracellular Vesicle Content and Internalization to Attenuate Extracellular Vesicle-Mediated Osteoclastogenesis and Osteoblast Proliferation. J Extracell Vesicles (2014) 3(1):23784. 10.3402/jev.v3.23784 PMC4072912 25018864

[212] Cao J Navis A Cox BD Dickson AL Gemberling M Karra R. Single Epicardial Cell Transcriptome Sequencing Identifies Caveolin 1 as an Essential Factor in Zebrafish Heart Regeneration. Development (2016) 143:232–43. 10.1242/dev.130534 PMC4725347 26657776

[213] Fernández-Hernando C Yu J Suárez Y Rahner C Dávalos A Lasunción MA. Genetic Evidence Supporting a Critical Role of Endothelial Caveolin-1 During the Progression of Atherosclerosis. Cell Metab (2009) 10:48–54. 10.1016/j.cmet.2009.06.003 19583953 PMC2735117

[214] Qian S Tan X Liu X Liu P Wu Y. Exosomal Tenascin-C Induces Proliferation and Invasion of Pancreatic Cancer Cells by WNT Signaling. Onco Targets Ther (2019) 12:3197–205. 10.2147/OTT.S192218 PMC6499136 31118672

[215] Daassi D Mahoney KM Freeman GJ. The Importance of Exosomal PDL1 in Tumour Immune Evasion. Nat Rev Immunol (2020) 20:209–15. 10.1038/s41577-019-0264-y 31965064

[216] Sur S Kaur H Roy S. Exosomes From COVID-19 Patients Carry Tenascin-C and Fibrinogen-β in Triggering Inflammatory Signals in Cells of Distant Organ. Int J Mol Sci (2021) 22. 10.1101/2021.02.08.430369 PMC8003878 33804769

[217] Li C Cui Y Luan J Zhou X Li H Wang H. Tenascin C Affects Mineralization of Saos2 Osteoblast-Like Cells Through Matrix Vesicles. Drug Discovery Ther (2016) 10:82–7. 10.5582/ddt.2016.01009 26961327

[218] Mills JT Schwenzer A Marsh EK Edwards MR Sabroe I Midwood KS. Airway Epithelial Cells Generate Pro-Inflammatory Tenascin-C and Small Extracellular Vesicles in Response to TLR3 Stimuli and Rhinovirus Infection. Front Immunol (2019) 10:1987. 10.3389/fimmu.2019.01987 31497021 PMC6712508

[219] Mirzaei R Sarkar S Dzikowski L Rawji KS Khan L Faissner A. Brain Tumor-Initiating Cells Export Tenascin-C Associated With Exosomes to Suppress T Cell Activity. Oncoimmunology (2018) 7:e1478647. 10.1080/2162402X.2018.1478647 30288344 PMC6169571

[220] Wei W Ao Q Wang X Cao Y Liu Y Zheng SG. Mesenchymal Stem Cell-Derived Exosomes: A Promising Biological Tool in Nanomedicine. Front Pharmacol (2020) 11:590470. 10.3389/fphar.2020.590470 33716723 PMC7944140

[221] Sharma P Kaur H Roy S. Designing a Tenascin-C-Inspired Short Bioactive Peptide Scaffold to Direct and Control Cellular Behavior. ACS Biomater Sci Eng (2019) 5:6497–510. 10.1021/acsbiomaterials.9b01115 33417802

[222] Rosenbloom J Macarak E Piera-Velazquez S Jimenez SA. Human Fibrotic Diseases: Current Challenges in Fibrosis Research. Methods Mol Biol (2017) 1627:1–23. 10.1007/978-1-4939-7113-8_1 28836191

[223] Zhao X Kwan JYY Yip K Liu PP Liu FF. Targeting Metabolic Dysregulation for Fibrosis Therapy. Nat Rev Drug Discov (2020) 19:57–75. 10.1038/s41573-019-0040-5 31548636

[224] Schuppan D Ashfaq-Khan M Yang AT Kim YO. Liver Fibrosis: Direct Antifibrotic Agents and Targeted Therapies. Matrix Biol (2018) 68–69:435–51. 10.1016/j.matbio.2018.04.006 29656147

[225] Meng XM Nikolic-Paterson DJ Lan HY. Tgf-β: The Master Regulator of Fibrosis. Nat Rev Nephrol (2016). 10.1038/nrneph.2016.48 27108839

[226] Somogyi V Chaudhuri N Torrisi SE Kahn N Müller V Kreuter M. the Therapy of Idiopathic Pulmonary Fibrosis: What is Next? Eur Respir Rev (2019) 28(153):190021. 10.1183/16000617.0021-2019 31484664 PMC9488691

[227] Maher TM Strek ME. Antifibrotic Therapy for Idiopathic Pulmonary Fibrosis: Time to Treat. Respir Res (2019) 20:205. 10.1186/s12931-019-1161-4 31492155 PMC6731623

[228] Hauge A Rofstad EK. Antifibrotic Therapy to Normalize the Tumor Microenvironment. J Trans Med (2020) 18(1):207. 10.1186/s12967-020-02376-y PMC7240990 32434573

